# [2+2+2] Annulation of *N*-(1-Naphthyl)acetamide with Two Alkynoates via Cleavage of Adjacent C–H and C–N Bonds Catalyzed by an Electron-Deficient Rhodium(III) Complex

**DOI:** 10.3390/molecules23123325

**Published:** 2018-12-14

**Authors:** Jyunichi Terasawa, Yu Shibata, Miho Fukui, Ken Tanaka

**Affiliations:** Department of Chemical Science and Engineering, Tokyo Institute of Technology, O-okayama, Meguro-ku, Tokyo 152-8550, Japan; terasawa.j.aa@m.titech.ac.jp (J.T.); mfv1.1.174@gmail.com (M.F.)

**Keywords:** alkynes, C–H bond cleavage, C–N bond cleavage, cyclopentadienyl complexes, *N*-(1-naphthyl)acetamide, rhodium, [2+2+2] annulation

## Abstract

It has been established that an electron-deficient cationic Cp^E^-rhodium(III) complex catalyzes the non-oxidative [2+2+2] annulation of *N*-(1-naphthyl)acetamide with two alkynoates via cleavage of the adjacent C–H and C–N bonds to give densely substituted phenanthrenes under mild conditions (at 40 °C under air). In this reaction, a dearomatized spiro compound was isolated, which may support the formation of a cationic spiro rhodacycle intermediate in the catalytic cycle. The use of *N*-(1-naphthyl)acetamide in place of acetanilide switched the reaction pathway from the oxidative [2+2+2] annulation-lactamization via C–H/C–H cleavage to the non-oxidative [2+2+2] annulation via C–H/C–N cleavage. This chemoselectivity switch may arise from stabilization of the carbocation in the above cationic spiro rhodacycle by the neighboring phenyl and acetylamino groups, resulting in the nucleophilic C–C bond formation followed by β-nitrogen elimination.

## 1. Introduction

**Scheme 1:**
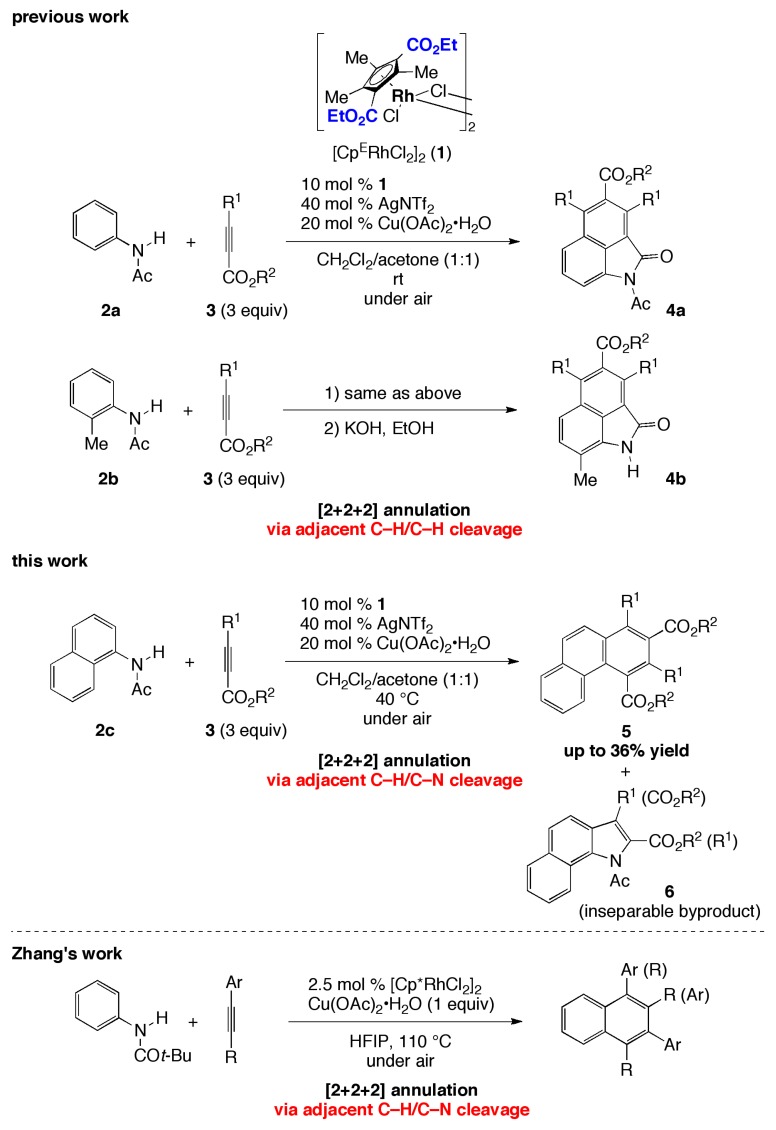
Cationic Cp^E^-rhodium(III) complex-catalyzed [2+2+2] annulations of acetanilides **2a**/**2b** and *N*-(1-naphthyl)acetamide (**2c**) with two alkynoates **3** via cleavage of adjacent C–H/C–H and C–H/C–N bonds, respectively.

The transition-metal-catalyzed [2+2+2] annulation of three unsaturated compounds is a useful method for the synthesis of six-membered carbocycles and heterocycles [1,2,3,4,5,6,7,8,9]. For example, the transition-metal-catalyzed [2+2+2] annulation of three alkynes is able to afford densely substituted benzenes with an atom- and step-economical manner [10,11,12,13,14]. For the synthesis of naphthalene derivatives, benzynes have been employed as one of the three alkynes but the use of the benzynes suffers from the redundant precursor synthesis and harsh reaction conditions [15,16,17]. Alternatively, several examples of the transition-metal-catalyzed oxidative [2+2+2] annulation via cleavage of adjacent two C–H bonds of benzenes, in which formally dehydrogenated benzenes are employed as benzyne equivalents, have been reported [18,19,20,21,22]. As such, our research group reported that an electron-deficient cationic Cp^E^-rhodium(III) complex, derived from **1**, catalyzes the oxidative tandem [2+2+2] annulation-lactamization of acetanilide (**2a**) with two alkynoates **3** via cleavage of adjacent two C–H bonds at room temperature under air to give densely substituted banzo[*cd*]indolones **4a** (Scheme 1, top) [23]. In this catalysis, not only acetanilide (**2a**) but also *ortho*-substituted acetanilide **2b** was able to react with two alkynoates **3** to give the corresponding banzo[*cd*]indolones **4b** after treatment with KOH in ethanol (Scheme 1, top) [23]. In this paper, we have established that the electron-deficient cationic Cp^E^-rhodium(III) complex is able to catalyze the non-oxidative [2+2+2] annulation of *N*-(1-naphthyl)acetamide (**2c**) with two alkynoates **3** via cleavage of the adjacent C–H and C–N bonds, leading to densely substituted phenanthrenes **5**, under mild conditions (at 40 °C under air) (Scheme 1, middle). Although several examples of the transition-metal-catalyzed decarboxylative and oxidative [2+2+2] annulation of benzoic acids with two alkynoates via cleavage of the adjacent C–H and C–C bonds, leading to densely substituted naphthalenes, have been reported [24,25,26,27], only a single example of the non-oxidative [2+2+2] annulation via cleavage of the adjacent C–H and C–N bonds, in which the acylamino moiety is employed as a traceless directing group, has been reported in the neutral Cp*-rhodium(III) complex-catalyzed synthesis of tetraarylnaphthalenes from *N*-acylanilines and two diarylacetylenes at elevated temperature (110 °C) (Scheme 1, bottom) [28].

## 2. Results

**Scheme 2:**
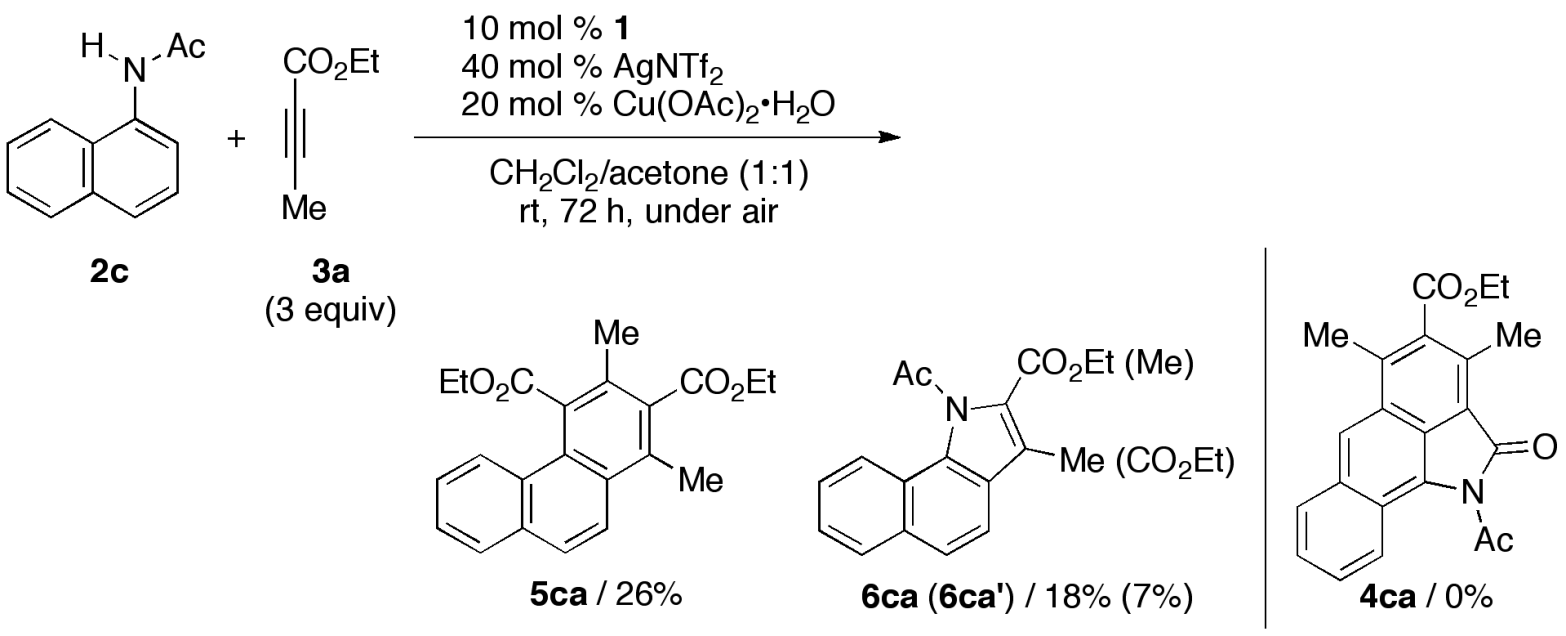
Cationic Cp^E^-rhodium(III) complex-catalyzed non-oxidative [2+2+2] annulation of *N*-(1-naphthyl)acetamide (**2c**) with two ethyl 2-butynoates (**3a**).

In the course of our study of the cationic Cp^E^-rhodium(III) complex-catalyzed oxidative tandem [2+2+2] annulation-lactamization of acetanilides with two alkynoates, leading to banzo[*cd*]indolones, the reaction of 2-methyl acetanilide **2b** and ethyl 2-butynoates (**3a**) was examined. As already shown in Scheme 1, the expected banzo[*cd*]indolones **4b** were generated after treatment with KOH in ethanol [23]. Surprisingly, the use of *N*-(1-naphthyl)acetamide (**2c**) in place of **2b** failed to afford the expected naphtho[*cd*]indolone **4ca**. Instead, densely substituted phenanthrene **5ca** was generated as a major product along with a mixture of the corresponding regioisomeric [3+2] annulation products **6ca**/**6ca′** (Scheme 2). In addition to the above products, unidentified oligomerization products derived from **2c** and **3a** were generated as by-products.

Then the screening of the reaction conditions and the acyl groups on the nitrogen of 1-aminonaphthalene was conducted as shown in Table 1. Elevating the reaction temperature (40 °C) slightly increased the yields of **5ca** and **6ca**/**6ca′** (entry 2). Increasing the amount of **3a** increased the yield of **5ca** and decreased that of **6ca**/**6ca′** (entry 3). However, this increase was very small, therefore, the conditions of entry 2 were selected as the best conditions. With respect to the acyl groups on the nitrogen of the 1-aminonaphthalene moiety, electron-poor *N*-(1-naphthyl)amide **2d**, possessing the highly acidic amide proton, was tested in place of **2c**, while no reaction was observed even at 80 °C (entry 4). Sterically demanding *N*-(1-naphthyl)amide **2e** was also tested with expectation for acceleration of reductive elimination. Unfortunately, the use of **2e** significantly increased the yield of not the [2+2+2] annulation product **5ea** but the [3+2] annulation products **6ea**/**6ea′** (entry 5). Finally, the reaction was conducted using the Cp*-rhodium(III) complex instead of the Cp^E^-rhodium(III) complex **1**, which resulted in significant decrease of the yields of **5ca** and **6ca**/**6ca′** (entry 6).

**Figure 1:**
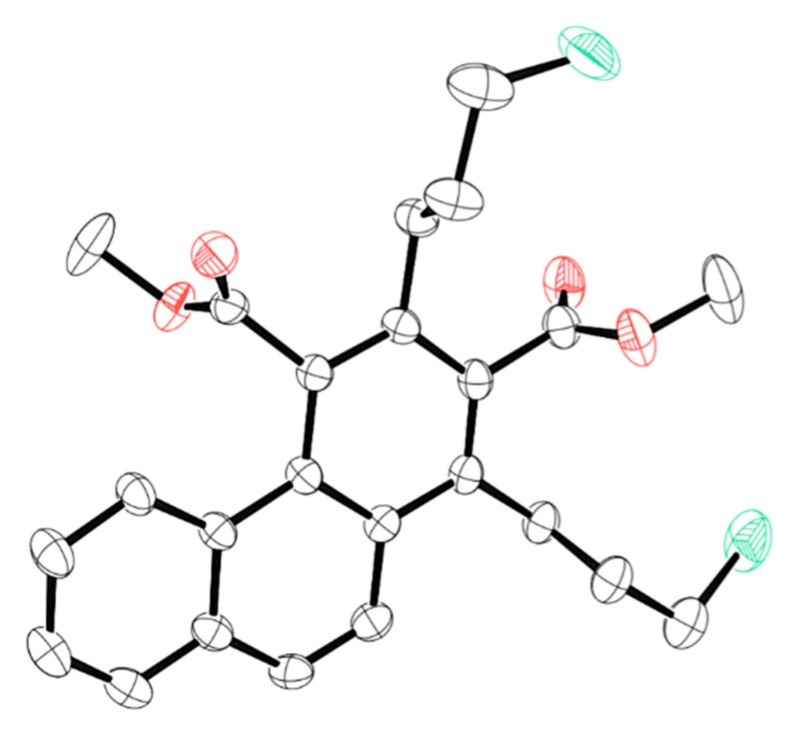
ORTEP diagram of **5ce** with ellipsoids at 50% probability. All hydrogen and disordered atoms are omitted for clarity.

The scope of the present cationic Cp^E^-rhodium(III) complex-catalyzed [2+2+2] annulation is shown in Table 2. As with our previously reported cationic Cp^E^-rhodium(III) complex-catalyzed oxidative tandem [2+2+2] annulation-lactamization of acetanilides with two alkynoates, leading to banzo[*cd*]indolones, a wide variety of primary alkyl-substituted alkynoates **3a**–**e** reacted with *N*-(1-naphthyl)acetamide (**2c**) to give the corresponding [2+2+2] annulation products **5ca**–**ce** (entries 1–5). However, phenyl-substituted alkynoate **3f** failed to afford the corresponding [2+2+2] annulation product **5cf** and gave the corresponding [3+2] annulation product **6cf′** in good yield with perfect regioselectivity (entry 6). This product switch is also the same as our previously reported tandem [2+2+2] annulation-lactamization of acetanilide (**2a**) with **3f** [23]. Unfortunately, the use electron-deficient alkynes did not afford the corresponding annulation products at all. For example, the use of ethyl propiolate afforded a complex mixture of products and no reaction was observed when using ethyl 2-butynoate. The structure and regiochemistry of the [2+2+2] annulation product **5ce** were unambiguously determined by the X-ray crystallographic analysis as shown in Figure 1.

**Scheme 3:**
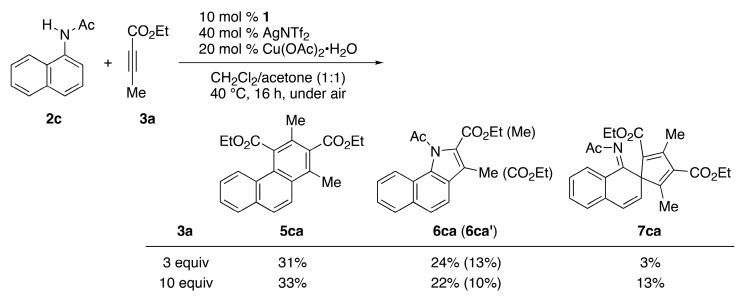
Formation of dearomatized spiro compound **7ca**. The yields were determined by ^1^H NMR yield using C_6_Me_6_ as an internal standard.

Importantly, an interesting by-product was generated in the reaction of **2c** and **3a** as shown in Scheme 3. When using 3 equiv of **3a**, a very small amount of dearomatized spiro compound **7ca** was detected in the crude reaction mixture (3% yield). An isolable amount (13% yield) of **7ca** was generated when using an excess amount of **3a**.

**Scheme 4:**
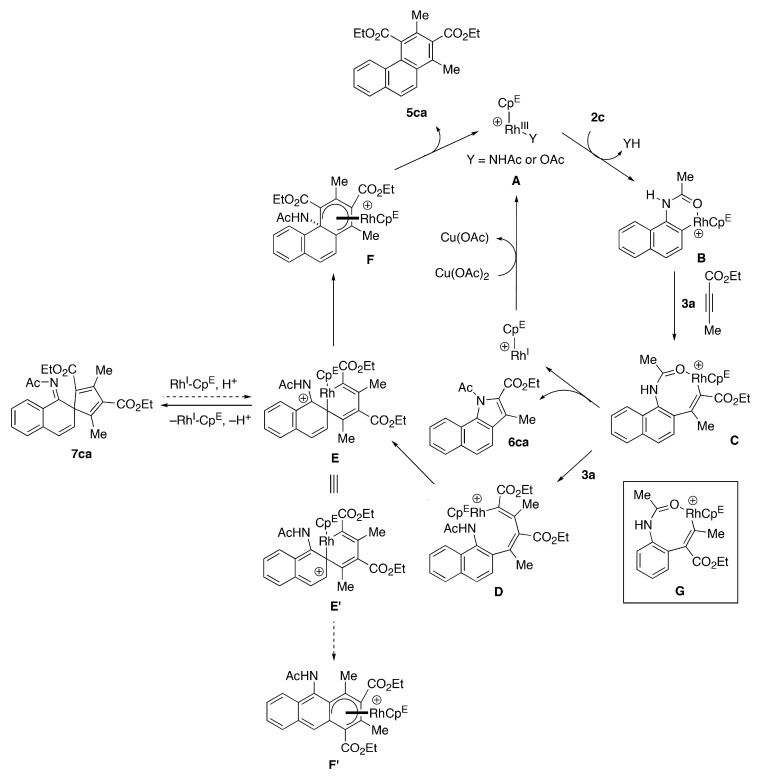
Plausible mechanisms for formation of **5ca**, **6ca** and **7ca** from **2c** and **3a**.

Plausible mechanisms for the formation of **5ca**, **6ca** and **7ca** from **2c** and **3a** are shown in Scheme 4. First, C–H bond cleavage of **2c** by Cp^E^-rhodium(III) species **A** affords naphthylrhodium **B**. Next, insertion of **3a** into **B** gives alkenylrhodium **C**. The subsequent insertion of **3a** into **C** gives dienylrhodium **D**. The second alkyne insertion may not proceed in the case of sterically demanding phenyl-substituted alkynoate **3f**, thus generating [3+2] annulation product **6cf′** (Table 2, entry 6). Electrophilic metalation of the electron-rich 1-aminonaphthalene ring produces cationic spiro rhodacycle **E**, in which the carbocation is stabilized by the neighboring phenyl and acetylamino groups. Nucleophilic attack of the dienylrhodium moiety to the electrophilic 1-position of the 1-aminonaphthalene ring gives π-pentadienyl complex **F** [29]. Importantly, nucleophilic attack of the dienylrhodium moiety to the 3-position of the 1-aminonaphthalene ring in spiro rhodacycle **E′**, leading to π-pentadienyl complex **F′**, would be unfavorable due to the unstable dearomatized quinodimethane structures in **E′** and **F′**. β-Nitrogen elimination [30,31] from intermediate **F** affords **5ca** and the catalytically active rhodium(III) species **A**. However, the copper(II) co-catalyst was necessary in order to reoxidize rhodium(I) species generated through the competing oxidative [3+2] annulation giving **6ca**. In this reaction, dearomatized spiro compound **7ca** was isolated as a by-product. This compound may be generated by reductive elimination and deprotonation from spiro rhodacycle **E**. Increasing the amount of **3a** may facilitate the reductive elimination, which increased the yield of **7ca**. This result may support the intermediacy of spiro rhodacycle **E** in the catalytic cycle. Interestingly, the regioselectivity of both the present Cp^E^-rhodium(III)-catalyzed [2+2+2] and [3+2] annulations of *N*-(1-naphthyl)acetamide (**2c**) is opposite to that of our previously reported Cp^E^-rhodium(III)-catalyzed [2+2+2] and [3+2] annulations of acetanilides that proceed presumably via alkenylrhodium **G** [23], although the reason is not clear at the present stage.

**Scheme 5:**
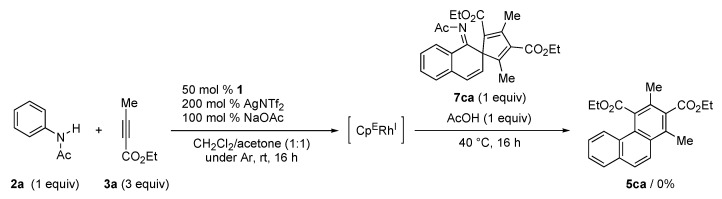
Reaction of **7ca** with an in situ generated Cp^E^-rhodium(III) complex and acetic acid.

We considered that it is possible to regenerate spiro rhodacycle **E** by oxidative addition with a neutral rhodium(I) complex and protonation of **7ca**. Therefore, **7ca** was treated with acetic acid and an in situ generated neutral Cp^E^-rhodium(I) complex, prepared by the reaction of **1**, AgNTf_2_, NaOAc, **2a** and **3a**. However, no reaction was observed, thus excluding the intermediacy of **7ca** in the catalytic cycle (Scheme 5).

## 3. Materials and Methods

### 3.1. General Information

Anhydrous acetone (No. 27,072-5) and CH_2_Cl_2_ (No. 130-02457) were obtained from Aldrich (St. Louis, MO, USA) and Wako (Osaka, Japan) and used as received. Solvents for the synthesis of substrates were dried over Molecular Sieves 4Å (Wako) prior to use. Anilides **2d** [32] and **2e** [33] were prepared according to the literature. Internal alkynes **3d** [34] and **3e** [35] were prepared according to the literature. All other reagents were obtained from commercial sources and used as received. ^1^H and ^13^C data were collected on a Bruker AVANCE III (Billerica, MA, USA) HD 400 (400 MHz) at ambient temperature. HRMS data were obtained on a Bruker micro TOF Focus II (Billerica, MA, USA). A single crystal X-ray diffraction measurement was made on Rigaku XtaLAB mini II diffractometer (Akishima, Japan) using graphite monochromated Mo-Kα radiation. All reactions were carried out in oven-dried glassware with magnetic stirring.

### 3.2. General Procedure for the Rhodium-Catalyzed Annulation of N-(1-Naphthyl)amides with Two Alkynoates (5ca, 6ca and 6ca′; Table 1, entry 2)

To a Schlenk tube was added AgNTf_2_ (15.5 mg, 0.040 mmol), [Cp^E^RhCl_2_]_2_ **1** (8.5 mg, 0.010 mmol), Cu(OAc)_2_·H_2_O (4.0 mg, 0.020 mmol), *N*-(naphthalen-1-yl)acetamide (**2c**, 18.5 mg, 0.100 mmol), ethyl 2-butynoate (**3a**, 33.6 mg, 0.300 mmol), acetone (0.5 mL) and CH_2_Cl_2_ (0.5 mL) under air in this order. The mixture was sealed and stirred at 40 °C under air for 16 hours. The resulting mixture was diluted with ether, filtered through a silica gel pad and washed with ether. The solvent was removed under reduced pressure and the residue was purified by a preparative thin layer chromatography (TLC, hexane/AcOEt = 2:1), to give a mixture of **5ca**, **6ca** and **6ca′**. The yields of **5ca** (31%), **6ca** (22%) and **6ca′** (10%) were determined by ^1^H NMR with hexamethylbenzene as an internal standard.

### 3.3. Product Characterization

*Diethyl 1,3-dimethylphenanthrene-2,4-dicarboxylate* (**5ca**). Analytically pure **5ca** was isolated from a mixture of **5ca**, **6ca** and **6ca′** (21.8 mg, **5ca**/**6ca**/**6ca′** = 45:41:14) by a gel permeation chromatography (GPC). The regiochemistry of the title compound was determined by the NOESY experiment as well as analogy to the ^1^H NMR chemical shifts of **5ce**. Colorless solid, 7.2 mg, 0.0205 mmol, 21% isolated yield, mp 83.8–85.0 °C; ^1^H NMR (CDCl_3_, 400 MHz) *δ* 8.44 (d, *J* = 8.4 Hz, 1H), 7.95 (d, *J* = 9.2 Hz, 1H), 7.92–7.87 (m, 1H), 7.79 (d, *J* = 9.2 Hz, 1H), 7.63–7.51 (m, 2H), 4.57–7.44 (m, 4H), 2.70 (s, 3H), 2.47 (s, 3H), 1.95 (s, 3H), 1.44 (t, *J* = 7.1 Hz, 3H), 1.35 (t, *J* = 7.2 Hz, 3H); ^13^C NMR (CDCl_3_, 100 MHz) *δ* 172.0, 170.0, 134.1, 133.0, 132.3, 129.8, 129.6, 129.5, 129.1, 128.7, 127.8, 127.5, 127.0, 126.3, 125.6, 122.4, 61.8, 61.4, 17.4, 17.2, 14.3, 13.9; HRMS (ESI) calcd for C_22_H_22_O_4_Na [M + Na]^+^ 373.1414, found 373.1410.

*Ethyl 1-acetyl-3-methyl-1H-benzo[g]indole-2-carboxylate* (**6ca**) *and ethyl 1-acetyl-2-methyl-1H-benzo[g]indole-3-carboxylate* (**6ca′**) [36]. An analytically pure mixture of **6ca** and **6ca′** was isolated from a mixture of **5ca**, **6ca** and **6ca′** (21.8 mg, **5ca**/**6ca**/**6ca′** = 45:41:14) by GPC. The regiochemistry of the title compounds was determined by comparison with the ^1^H NMR chemical shifts of the literature. Colorless solid, 7.6 mg, 0.0257 mmol, 26% isolated yield, **6ca**/**6ca′** = 75:25, mp 86.1–87.2 °C; ^1^H NMR (CDCl_3_, 400 MHz) *δ* **6ca**: 8.17–8.11 (m, 1H), 7.96–7.90 (m, 1H), 7.67 (d, *J* = 8.7 Hz, 1H), 7.59 (d, *J* = 8.7 Hz, 1H), 7.55–7.48 (m, 2H), 4.43 (q, *J* = 7.1 Hz, 2H), 2.76 (s, 3H), 2.65 (s, 3H), 2.04 (s, 3H), 1.45 (t, *J* = 7.1 Hz, 3H); **6ca′**: 8.28 (d, *J* = 8.7 Hz, 1H), 7.97 (d, *J* = 7.9 Hz, 1H), 7.74 (t, *J* = 7.9 Hz, 1H), 7.57–7.44 (m, 3H), 4.50–4.40 (m, 2H), 2.85 (s, 3H), 2.62 (s, 3H), 1.49 (t, *J* = 7.1 Hz, 3H); ^13^C NMR (CDCl_3_, 100 MHz) *δ* 177.6, 162.3, 133.5, 129.41, 129.36, 126.3, 126.2, 125.8, 125.2, 124.7, 124.4, 124.0, 123.7, 123.2, 121.8, 121.7, 121.3, 120.8, 119.0, 61.2, 30.0, 14.3, 10.4; HRMS (ESI) calcd for C_17_H_15_NONa [M + Na]^+^ 318.1101, found 318.1115.

*Dimethyl 1,3-dimethylphenanthrene-2,4-dicarboxylate* (**5cb**). The yield of **5cb** (30%) was determined by ^1^H NMR with hexamethylbenzene as an internal standard. Analytically pure **5cb** was isolated from a mixture of **5cb**, **6cb** and **6cb′** (19.2 mg, **5cb**/**6cb**/**6cb′** = 51:37:12) by GPC. The regiochemistry of the title compound was determined by analogy to the ^1^H NMR chemical shifts of **5ce**. Colorless solid, 8.8 mg, 0.0273 mmol, 27% isolated yield, mp 62.8–64.5 °C; ^1^H NMR (CDCl_3_, 400 MHz); ^1^H NMR (CDCl_3_, 400 MHz) *δ* 8.35 (d, *J* = 8.4 Hz, 1H), 7.95 (d, *J* = 9.1 Hz, 1H), 7.93–7.87 (m, 1H), 7.79 (d, *J* = 9.2 Hz, 1H), 7.64–7.53 (m, 2H), 4.00 (s, 3H), 3.99 (s, 3H), 2.68 (s, 3H), 2.44 (s, 3H); ^13^C NMR (CDCl_3_, 100 MHz) *δ* 172.4, 170.5, 133.9, 133.1, 132.6, 129.83, 129.79, 129.1, 129.0, 128.8, 127.9, 127.7, 127.1, 126.4, 125.3, 122.3, 52.7, 52.3 17.5, 17.4; HRMS (ESI) calcd for C_20_H_18_O_4_Na [M + Na]^+^ 345.1097, found 345.1101.

*Methyl 1-acetyl-3-methyl-1H-benzo[g]indole-2-carboxylate* (**6cb**) *and methyl 1-acetyl-2-methyl-1H-benzo[g]indole-3-carboxylate* (**6cb′**). The yields of **6cb** (22%) and **6cb′** (8%) was determined by ^1^H NMR with hexamethylbenzene as an internal standard. An analytically pure mixture of **6cb** and **6cb′** was isolated from a mixture of **5cb**, **6cb** and **6cb′** (19.2 mg, **5cb**/**6cb**/**6cb′** = 51:37:12) by GPC. The regiochemistry of the title compounds was determined by analogy to the ^1^H NMR chemical shifts of **6ca** and **6ca′**. Colorless solid, 6.0 mg, 0.0213 mmol, 21% isolated yield, **6cb**/**6cb′** = 75:25, mp 93.7–95.0 °C; ^1^H NMR (CDCl_3_, 400 MHz) **6cb**: *δ* 8.17–8.10 (m, 1H), 7.95–7.89 (m, 1H), 7.66 (d, *J* = 8.7 Hz, 1H), 7.59 (d, *J* = 8.7 Hz, 1H), 7.57–7.44 (m, 2H), 3.97 (s, 3H), 2.76 (s, 3H), 2.64 (s, 3H); **6cb′**: *δ* 8.26 (d, *J* = 8.8 Hz, 1H), 7.97 (d, *J* = 8.1 Hz, 1H), 7.93–7.87 (m, 1H), 7.79 (d, *J* = 9.2 Hz, 1H), 7.64–7.53 (m, 2H), 4.00 (s, 3H), 3.99 (s, 3H), 2.68 (s, 3H), 2.44 (s, 3H); ^13^C NMR (CDCl_3_, 100 MHz) *δ* 171.0, 136.8, 134.9, 133.6, 130.3, 130.2, 128.7, 128.5, 125.3, 123.4, 118.6, 118.1, 116.3, 27.6, 9.2; HRMS (ESI) calcd for C_17_H_15_NONa [M + Na]^+^ 304.0944, found 304.0952.

*Diethyl 1,3-dibutylphenanthrene-2,4-dicarboxylate* (**5cc**). The yield of **5cc** (32%) was determined by ^1^H NMR with hexamethylbenzene as an internal standard. Analytically pure **5cc** was isolated from a mixture of **5cc**, **6cc** and **6cc′** (40.0 mg, **5cc**/**6cc**/**6cc′** = 42:9:49) by GPC. The regiochemistry of the title compound was determined by analogy to the ^1^H NMR chemical shifts of **5ce**. Pale yellow oil, 11.7 mg, 0.0269 mmol, 27% isolated yield; ^1^H NMR (CDCl_3_, 400 MHz) *δ* 8.42 (d, *J* = 8.6 Hz, 1H), 7.93 (d, *J* = 9.2 Hz, 1H), 7.90–7.86 (m, 1H), 7.77 (d, *J* = 9.1 Hz, 1H), 7.61–7.56 (m, 1H), 7.55–7.50 (m, 1H), 4.51–4.43 (m, 4H), 3.04–2.97 (m, 2H), 2.80–2.68 (m, 2H), 1.78–1.64 (m, 4H), 1.55–1.29 (m, 4H), 1.44 (t, *J* = 7.1 Hz, 3H), 1.34 (t, *J* = 7.2 Hz, 3H), 1.02–0.92 (m, 6H); ^13^C NMR (CDCl_3_, 100 MHz) *δ* 171.9, 170.0, 137.5, 135.0, 133.7, 132.9, 129.4, 129.2, 129.1, 128.5, 128.1, 127.7, 127.0, 126.1, 125.7, 122.3, 61.7, 61.3, 33.9, 33.5, 32.1, 31.3, 23.4, 23.3, 14.2, 13.9, 13.83, 13.79; HRMS (ESI) calcd for C_28_H_34_O_4_Na [M + Na]^+^ 457.2349, found 457.2359.

*Ethyl 1-acetyl-3-butyl-1H-benzo[g]indole-2-carboxylate* (**6cc**) *and ethyl 1-acetyl-2-butyl-1H-benzo[g]indole-3-carboxylate* (**6cc′**). The yields of **6cc** (37%) and **6cc′** (7%) were determined by ^1^H NMR with hexamethylbenzene as an internal standard. An analytically pure mixture of **6cc** and **6cc′** was isolated from a mixture of **5cc**, **6cc** and **6cc′** (40.0 mg, **5cc**/**6cc**/**6cc′** = 42:9:49) by GPC. The regiochemistry of the title compounds was determined by analogy to the ^1^H NMR chemical shifts of **6ca** and **6ca′**. Colorless solid, 13.2 mg, 0.0391 mmol, 39% isolated yield, **6cc**/**6cc′** = 86:14, mp 61.0–62.4 °C; ^1^H NMR (CDCl_3_, 400 MHz) *δ* **6cc**: 8.16–8.09 (m, 1H), 7.95–7.88 (m, 1H), 7.67 (d, *J* = 8.7 Hz, 1H), 7.58 (d, *J* = 8.6 Hz, 1H), 7.56–7.43 (m, 2H), 4.43 (q, *J* = 7.1 Hz, 2H), 3.11 (t, *J* = 7.7 Hz, 2H), 2.77 (s, 3H), 1.75–1.63 (m, 2H), 1.52–1.40 (m, 2H), 1.44 (t, *J* = 7.1 Hz, 3H), 0.97 (t, *J* = 7.2 Hz, 3H); **6cc′**: 8.30 (d, *J* = 8.8 Hz, 1H), 8.00–7.95 (m, 1H), 7.77–7.71 (m, 2H), 7.56–7.43 (m, 2H), 4.50–4.39 (m, 2H), 3.25–3.18 (m, 2H), 1.75–1.63 (m, 2H), 1.52–1.40 (m, 5H), 1.00–0.93 (m, 3H); ^13^C NMR (CDCl_3_, 100 MHz) *δ* 177.7, 162.2, 133.4, 131.0, 129.4, 129.0, 126.3, 125.7, 124.8, 123.4, 123.1, 121.9, 121.6, 119.1, 61.2, 33.4, 30.0, 24.7, 22.9, 14.2, 14.0; HRMS (ESI) calcd for C_21_H_23_NO_3_Na [M + Na]^+^ 360.1570, found 360.1575.

*Dimethyl 1,3-bis(3-phenylpropyl)phenanthrene-2,4-dicarboxylate* (**5cd**). The yield of **5cd** (35%) was determined by ^1^H NMR with hexamethylbenzene as an internal standard. Analytically pure **5cd** was isolated from a mixture of **5cd**, **6cd** and **6cd′** (42.3 mg, **5cd**/**6cd**/**6cd′** = 46:7:47) by GPC. The regiochemistry of the title compound was determined by analogy to the ^1^H NMR chemical shifts of **5ce**. Pale yellow solid, 16.4 mg, 0.0294 mmol, 29% isolated yield, mp 91.0–92.6 °C; ^1^H NMR (CDCl_3_, 400 MHz) *δ* 8.30 (d, *J* = 8.4 Hz, 1H), 7.89–7.83 (m, 1H), 7.78–7.68 (m, 2H), 7.61–7.44 (m, 2H), 7.36–7.14 (m, 10H), 3.84 (s, 3H), 3.65 (s, 3H), 3.00–2.90 (m, 2H), 2.78 (t, *J* = 7.2 Hz, 2H), 2.70 (t, *J* = 7.2 Hz, 2H), 2.70–2.62 (m, 2H), 2.10–1.94 (m, 4H); ^13^C NMR (CDCl_3_, 100 MHz) *δ* 172.2, 170.1, 141.8, 141.6, 137.3, 134.6, 133.5, 132.9, 129.3, 129.2, 128.9, 128.7, 128.6, 128.4, 128.3, 128.2, 128.0, 127.1 126.3, 126.0, 125.8, 125.3, 122.1, 52.5, 52.0, 36.4, 36.2, 33.2, 32.6, 32.0, 30.9; HRMS (ESI) calcd for C_36_H_34_O_4_Na [M + Na]^+^ 553.2349, found 553.2354.

*Ethyl 1-acetyl-3-(3-phenylpropyl)-1H-benzo[g]indole-2-carboxylate* (**6cd**) *and ethyl 1-acetyl-2-(3-phenylpropyl)-1H-benzo[g]indole-3-carboxylate* (**6cd′**). The yields of **6cd** (38%) and **6cd′** (5%) were determined by ^1^HNMR with hexamethylbenzene as an internal standard. An analytically pure mixture of **6cd** and **6cd′** was isolated from a mixture of **5cd**, **6cd** and **6cd′** (42.3 mg, **5cd**/**6cd**/**6cd′** = 46:7:47) by GPC. The regiochemistry of the title compounds was determined by analogy to the ^1^H NMR chemical shifts of **6ca** and **6ca′**. Pale yellow oil, 14.1 mg, 0.0353 mmol, 35% isolated yield, **6cd**/**6cd′** = 89:11; ^1^H NMR (CDCl_3_, 400 MHz) *δ* 8.15–8.08 (m, 1H), 7.95–7.87 (m, 1H), 7.61–7.54 (m, 2H), 7.53–7.48 (m, 2H), 7.33–7.26 (m, 2H), 7.25–7.15 (m, 3H), 3.85 (s, 3H), 3.18–3.10 (m, 2H), 2.78–2.72 (m, 2H), 2.76 (s, 3H), 2.07–1.97 (m, 2H); ^13^C NMR (CDCl_3_, 100 MHz) *δ* 177.6, 162.6, 142.1, 133.4, 131.1, 129.4, 128.5, 128.3, 126.3, 125.84, 125.80, 124.7, 123.3, 123.2, 121.8, 121.6, 119.0, 51.9, 35.9, 32.5, 29.9, 24.5; HRMS (ESI) calcd for C_36_H_34_O_4_Na [M + Na]^+^ 408.1570, found 408.1574.

*Diethyl 1,3-dibutylphenanthrene-2,4-dicarboxylate* (**5ce**). The yield of **5ce** (36%) was determined by ^1^H NMR with hexamethylbenzene as an internal standard. Analytically pure **5ce** was isolated from a mixture of **5ce**, **6ce** and **6ce′** (42.6 mg, **5ce**/**6ce**/**6ce′** = 50:7:43) by GPC. The regiochemistry of the title compound was determined by the X-ray crystallographic analysis as shown in Figure 1. Colorless solid, 13.5 mg, 0.0284 mmol, 28% isolated yield, mp 108.1–109.2 °C; ^1^H NMR (CDCl_3_, 400 MHz) *δ* 8.34 (d, *J* = 8.4 Hz, 1H), 7.97 (d, *J* = 9.2 Hz, 1H), 7.93–7.88 (m, 1H), 7.82 (d, *J* = 9.2 Hz, 1H), 7.65–7.53 (m, 2H), 4.02 (s, 3H), 3.98 (s, 3H), 3.68 (t, *J* = 6.1 Hz, 2H), 3.63 (t, *J* = 6.1 Hz, 2H), 3.24–3.16 (m, 2H), 2.93–2.84 (m, 2H), 2.26–2.13 (m, 4H); ^13^C NMR (CDCl_3_, 100 MHz) *δ* 172.0, 170.0, 136.1, 133.9, 133.3, 133.0, 129.6, 129.5, 129.2, 128.8, 128.6, 128.4, 127.4, 126.6, 125.3, 121.8, 52.8, 52.5, 45.2, 45.0, 34.4, 33.7, 30.1, 28.9; HRMS (ESI) calcd for C_24_H_24_Cl_2_O_4_Na [M + Na]^+^ 469.0944, found 469.0948.

*Methyl 1-acetyl-3-(3-chloropropyl)-1H-benzo[g]indole-2-carboxylate* (**6ce**) *and methyl 1-acetyl-2-(3-chloropropyl)-1H-benzo[g]indole-3-carboxylate* (**6ce′**). The yields of **6ce** (31%) and **6ce′** (5%) were determined by ^1^H NMR with hexamethylbenzene as an internal standard. An analytically pure mixture of **6ce** and **6ce′** was isolated from a mixture of **5ce**, **6ce** and **6ce′** (42.6 mg, **5ce**/**6ce**/**6ce′** = 50:7:43) by GPC. The regiochemistry of the title compounds was determined by analogy to the ^1^H NMR chemical shifts of **6ce** and **6ce′**. Colorless oil, 8.6 mg, 0.0240 mmol, 24% isolated yield, **6ce**/**6ce′** = 83:17. ^1^H NMR (CDCl_3_, 400 MHz) **6ce**: *δ* 8.14–8.07 (m, 1H), 7.96–7.89 (m, 1H), 7.71 (d, *J* = 8.8 Hz, 1H), 7.60 (d, *J* = 8.8 Hz, 1H), 7.58–7.46 (m, 2H), 3.98 (s, 3H), 3.61 (t, *J* = 6.3 Hz, 2H), 3.32–3.26 (m, 2H), 2.79 (s, 3H), 2.30–2.13 (m, 2H); **6ce′**: *δ* 8.76 (d, *J* = 8.8 Hz 1H), 8.00–7.97 (m, 1H), 7.77–7.75 (m, 1H), 7.75–7.73 (m, 1H), 7.58–7.46 (m, 2H), 4.00 (s, 3H), 3.68 (t, *J* = 6.6 Hz, 2H), 3.37–3.32 (m, 2H), 2.62 (s, 3H), 2.30–2.13 (m, 2H); ^13^C NMR (CDCl_3_, 100 MHz) *δ* 177.5, 162.3, 133.5, 131.1, 129.5, 126.8, 126.5, 126.0, 125.0, 124.6, 123.5, 123.3, 121.7, 121.60, 120.8, 118.8, 52.1, 44.7, 33.7, 29.9, 22.1; HRMS (ESI) calcd for C_19_H_18_O_3_NClNa [M + Na]^+^ 366.0867, found 366.0871.

*Ethyl 2-phenyl-1-pivaloyl-1H-benzo[g]indole-3-carboxylate* (**6cf′**). The yield of **6cf′** (69%) was determined by ^1^H NMR with hexamethylbenzene as an internal standard. Analytically pure **6cf′** was isolated by repeated preparative TLCs. Pale yellow oil, 21.9 mg, 0.0612 mmol, 61% isolated yield; ^1^H NMR (CDCl_3_, 400 MHz) *δ* 8.37 (d, *J* = 8.8 Hz, 1H), 8.00–7.95 (m, 1H), 7.91–7.86 (m, 1H), 7.78 (d, *J* = 8.7 Hz, 1H), 7.55–7.44 (m, 7H), 4.22 (q, *J* = 7.1 Hz, 2H), 2.27 (s, 3H), 1.16 (t, *J* = 7.1 Hz, 3H), 1.17 (s, 9H); ^13^C NMR (CDCl_3_, 100 MHz) *δ* 176.0, 164.5, 142.4, 132.0, 131.2, 130.8, 129.43, 129.41, 129.3, 128.0, 126.1, 125.2, 125.0, 124.7, 121.7, 121.3, 120.6, 110.1, 59.9, 29.3, 14.0; HRMS (ESI) calcd for C_23_H_19_NO_3_Na [M + Na]^+^ 380.1257, found 380.1266.

*Ethyl 3-methyl-1-pivaloyl-1H-benzo[g]indole-2-carboxylate* (**6ea**) *and ethyl 2-methyl-1-pivaloyl-1H-benzo[g]indole-3-carboxylate* (**6ea′**). The yields of **6ea** (55%) and **6ea′** (3%) were determined by ^1^H NMR with hexamethylbenzene as an internal standard. An analytically pure mixture of **6ea** and **6ea′** was isolated by repeated preparative TLCs. Colorless solid; 15.5 g, 0.0459 mmol, 46% isolated yield, **6ea**. mp 128.4–129.5 °C; ^1^H NMR (CDCl_3_, 400 MHz) *δ* 8.24–8.17 (m, 1H), 7.91–7.86 (m, 1H), 7.66 (d, *J* = 8.7 Hz, 1H), 7.56 (d, *J* = 8.7 Hz, 1H), 7.52–7.42 (m, 2H), 4.42 (q, *J* = 7.1 Hz, 2H), 2.66 (s, 3H), 1.43 (t, *J* = 7.1 Hz, 3H), 1.17 (s, 9H); ^13^C NMR (CDCl_3_, 100 MHz) *δ* 187.5, 162.9, 133.4, 131.3, 129.1, 125.8, 125.7, 125.1, 124.7, 122.9, 122.8, 122.4, 122.3, 119.0, 61.2, 46.8, 28.2, 14.3, 10.4; HRMS (ESI) calcd for C_21_H_23_NO_3_Na [M + Na]^+^ 360.1570, found 360.1570.

*Diethyl (Z)-1′-(acetylimino)-3,5-dimethyl-1′H-spiro[cyclopentane-1,2′-naphthalene]-2,4-diene-2,4-dicarboxylate* (**7ca**). The yield of **7ca** (13%) was determined by ^1^H NMR with hexamethylbenzene as an internal standard. Analytically pure **7ca** was isolated by repeated preparative TLCs. The structure was determined by the HSQC and HMBC analyses. Pale yellow oil, 9.7 mg, 0.214 mmol, 11% isolated yield; ^1^H NMR (CDCl_3_, 400 MHz) *δ* 8.08 (d, *J* = 8.0 Hz, 1H, H*^h^*), 7.48 (td, *J* = 7.5, 1.3 Hz, 1H, H*^g^*), 7.31 (td, *J* = 7.7, 1.3 Hz, 1H, H*^f^*), 7.28–7.22 (m, 1H, H*^e^*), 6.79 (d, *J* = 9.4 Hz, 1H, H*^d^*), 5.05 (d, *J* = 9.4 Hz, 1H, H*^c^*), 4.31 (q, *J* = 7.1 Hz, 2H, CO_2_CH_2_CH_3_ on C^2^), 4.04 (q, *J* = 7.1 Hz, 2H, CO_2_CH_2_CH_3_ on C^4^), 2.50 (s, 3H, CH_3_ on C^3^), 2.10 (s, 3H, CH_3_ on C^1^), 2.01 (s, 3H, NCOCH_3_), 1.36 (t, *J* = 7.1 Hz, 3H, CO_2_CH_2_CH_3_ on C^2^), 0.89 (t, *J* = 7.1 Hz, 3H, CO_2_CH_2_CH_3_ on C^4^); ^13^C NMR (CDCl_3_, 100 MHz) *δ* 180.6 (NCOMe), 164.1 (C*^a^*), 164.0 (CO_2_Et), 163.0 (CO_2_Et), 158.7, 154.0 (C*^e^*), 136.0 (C*^j^*), 135.1, 132.6 (C*^h^*), 132.2, 129.9, 129.0 (C*^c^*), 127.9 (C*^g^*), 127.8 (C*^f^*), 127.5 (C*^d^*), 127.1 (C*^i^*), 67.7 (C*^b^*), 60.8 (CO_2_CH_2_CH_3_ on C^2^), 60.1 (CO_2_CH_2_CH_3_ on C^4^), 24.5 (NCOCH_3_), 15.1 (CH_3_ on C^3^), 14.2 (CO_2_CH_2_CH_3_ on C^2^), 13.9 (CH_3_ on C^1^), 13.5 (CO_2_CH_2_CH_3_ on C^4^); HRMS (ESI) calcd for C_24_H_25_NO_5_Na [M + Na]^+^ 430.1625, found 430.1623.

The ^1^H and ^13^C-NMR spectra of **5ca**–**5ce**, **6ca**–**6ce**, **6ca′**–**6cf′** and **7ca**, and crystal data and data collection parameters of **5ce** are available in Supplementary Materials.

## 4. Conclusions

In summary, we have established that an electron-deficient cationic Cp^E^-rhodium(III) complex catalyzes the non-oxidative [2+2+2] annulation of *N*-(1-naphthyl)acetamide with two alkynoates via cleavage of the adjacent C–H and C–N bonds to give densely substituted phenanthrenes under mild conditions (at 40 °C under air). Importantly, a dearomatized spiro compound was isolated in this reaction, which may support the formation of a spiro rhodacycle intermediate in the catalytic cycle. The use of *N*-(1-naphthyl)acetamide in place of acetanilide switched the reaction pathway from the oxidative tandem [2+2+2] annulation-lactamization involving cleavage of adjacent two C–H bonds to the non-oxidative [2+2+2] annulation involving cleavage of the adjacent C–H and C–N bonds. This chemoselectivity switch may arise from stabilization of the carbocation in the above cationic spiro rhodacycle by the neighboring phenyl and acetylamino groups, resulting in nucleophilic attack of the dienylrhodium moiety to this carbocation followed by β-nitrogen elimination.

## Figures and Tables

**Table 1:**
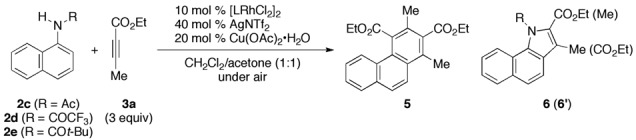
Optimization of reaction conditions for rhodium (III)-catalyzed [2+2+2] annulation of *N*-(1-naphthyl)amides **2** with two ethyl 2-butynoates (**3a**) *^a^*.

Entry	L	2 (R)	Conditions	3a (Equiv.)	Yield (%) *^b^*	
					5	6 (6′)
1	Cp^E^	**2c** (Ac)	rt, 72 h	3	**5ca**/26	**6ca** (**6ca′**)/18 (7)
2	Cp^E^	**2c** (Ac)	40 °C, 16 h	3	**5ca**/31	**6ca** (**6ca′**)/24 (13)
3	Cp^E^	**2c** (Ac)	40 °C, 16 h	10	**5ca**/33	**6ca** (**6ca′**)/22 (10)
4 *^c^*	Cp^E^	**2d** (COCF_3_)	80 °C, 16 h	3	**5da**/0	**6da** (**6da′**)/0 (0)
5	Cp^E^	**2e** (CO*t*-Bu)	40 °C, 16 h	3	**5ea**/0	**6ea** (**6ea′**)/55 (3)
6	Cp*	**2c** (Ac)	40 °C, 16 h	3	**5ca**/13	**6ca** (**6ca′**)/3 (<1)

*^a^* [Rh_2_] (0.020 mmol), AgNTf_2_ (0.080 mmol), Cu(OAc)_2_·H_2_O (0.040 mmol), **2** (0.200 mmol), **3a** (0.60–2.00 mmol), CH_2_Cl_2_ (1.0 mL) and acetone (1.0 mL) were used. *^b^* Determined by ^1^H NMR yield using C_6_Me_6_ as an internal standard. *^c^* (CH_2_Cl)_2_ (2.0 mL) was used.

**Table 2:**
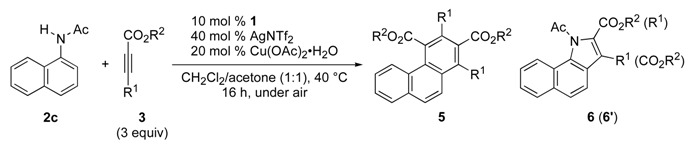
Scope of alkynoates **3** on rhodium(III)-catalyzed [2+2+2] annulation with *N*-(1-naphthyl)acetamide (**2c**) *^a^*.

Entry	3	R^1^	R^2^	Yield (%) *^b^*	
				5	6 (6′)
1	**3a**	Me	Et	**5ca**/33	**6ca** (**6ca′**)/22 (10)
2	**3b**	Me	Me	**5cb**/30	**6cb** (**6bb′**)/22 (8)
3	**3c**	*n*-Bu	Et	**5cc**/32	**6cc** (**6cc′**)/37 (7)
4	**3d**	Ph(CH_2_)_3_	Me	**5cd**/35	**6cd** (**6cd′**)/38 (5)
5	**3e**	Cl(CH_2_)_3_	Me	**5ce**/36	**6ce** (**6ce′**)/31 (5)
6	**3f**	Ph	Et	**5cf**/0	**6cf** (**6cf′**)/0 (69)

*^a^***1** (0.020 mmol), AgNTf_2_ (0.080 mmol), Cu(OAc)_2_·H_2_O (0.040 mmol), **2c** (0.200 mmol), **3** (0.600 mmol), CH_2_Cl_2_ (1.0 mL) and acetone (1.0 mL) were used. *^b^* Determined by ^1^H NMR yield using C_6_Me_6_ as an internal standard.

## Supplementary Materials

The following are available online: ^1^H and ^13^C NMR spectra of **5ca**–**5ce**, **6ca**–**6ce**, **6ca′**–**6cf′** and **7ca** and crystal data and data collection parameters of **5ce**.
