# Supplementary material for: [2+2+2] Annulation of N-(1-Naphthyl)acetamide with Two Alkynoates via Cleavage of Adjacent C–H and C–N Bonds Catalyzed by an Electron-Deficient Rhodium(III) Complex
Source: Molecules. 2018 Dec 14;23(12):3325. doi: 10.3390/molecules23123325 (PMC6321495; doi:10.3390/molecules23123325)

Supporting Information: ^{1}H and ^{13}C NMR Spectra

Diethyl 1,3-dimethylphenanthrene-2,4-dicarboxylate (5ca)

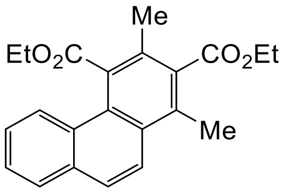

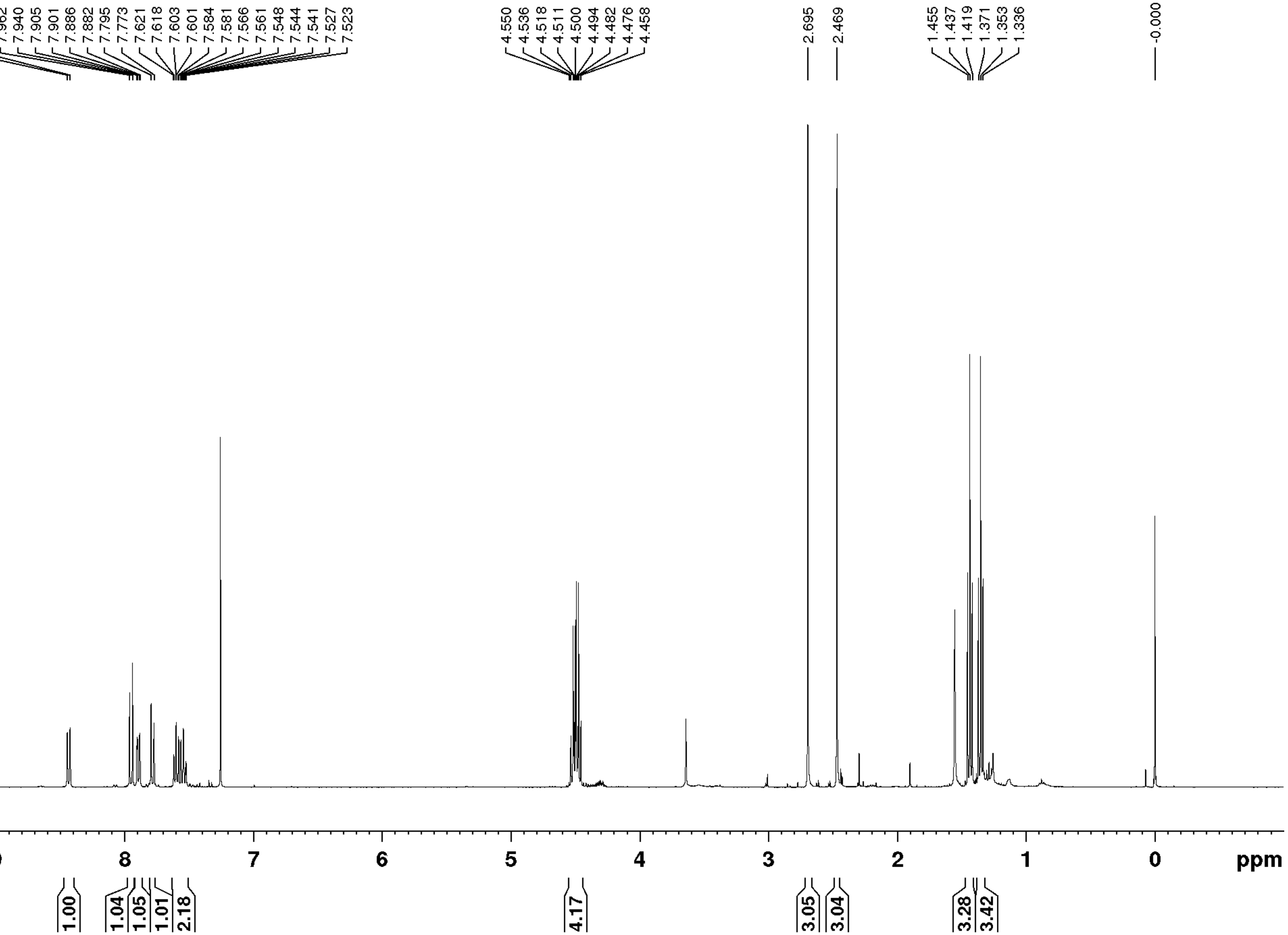

Diethyl 1,3-dimethylphenanthrene-2,4-dicarboxylate (5ca)

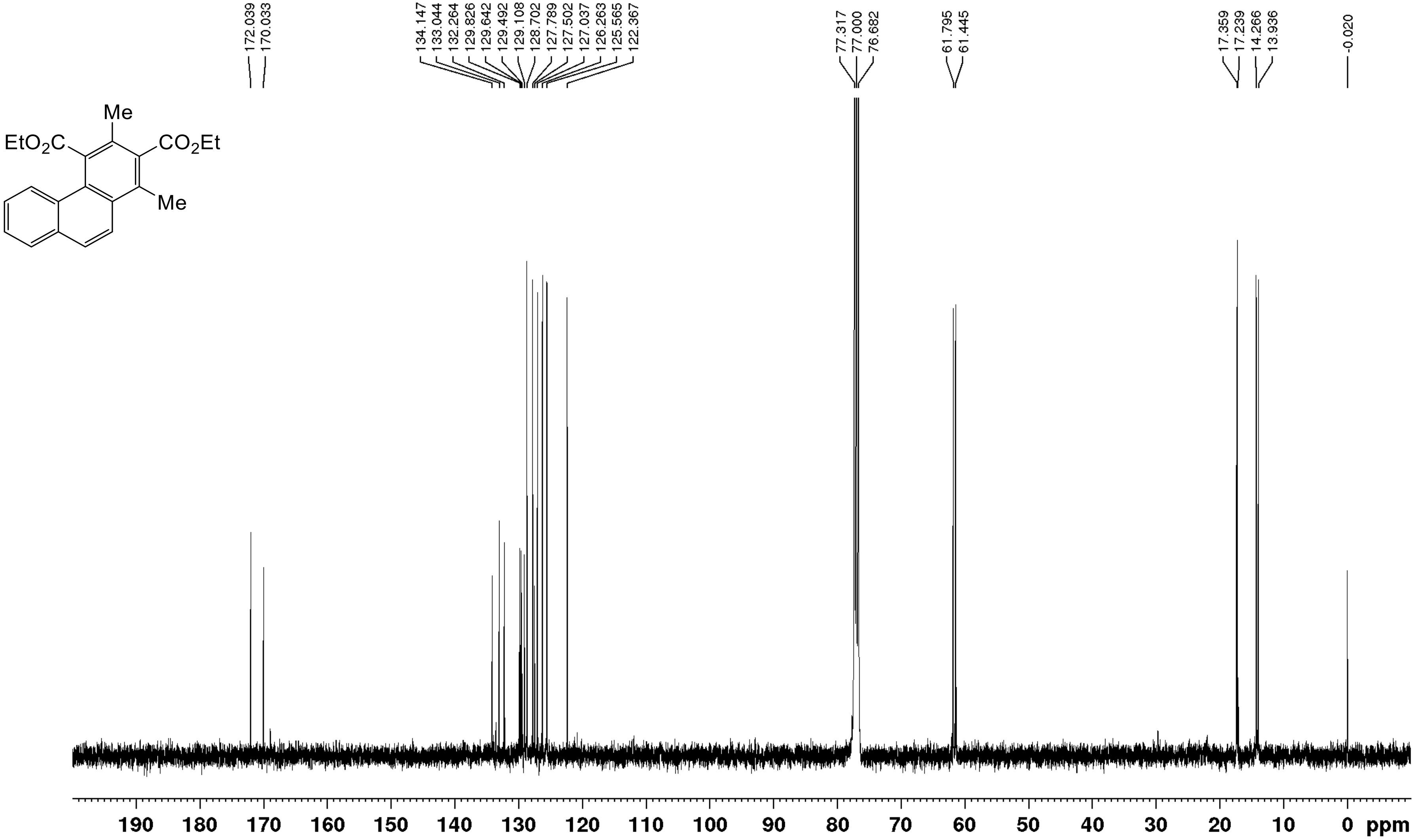

Ethyl 1-acetyl-3-methyl-1*H*-benzo[*g*]indole-2-carboxylate (6ca) and ethyl 1-acetyl-2-methyl-1*H*-benzo[*g*]indole-3-carboxylate (6ca')

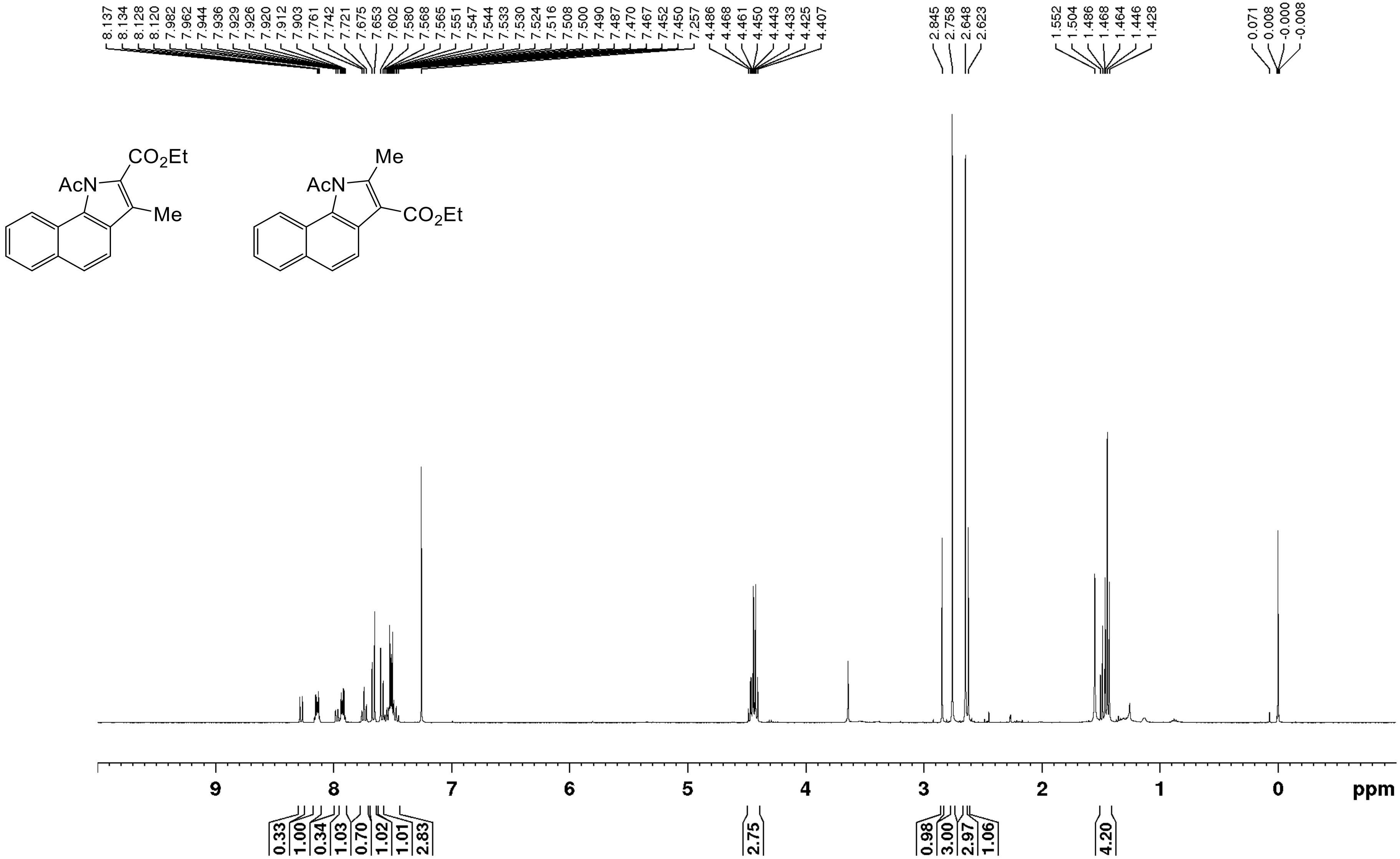

Ethyl 1-acetyl-3-methyl-1*H*-benzo[*g*]indole-2-carboxylate (6ca) and ethyl 1-acetyl-2-methyl-1*H*-benzo[*g*]indole-3-carboxylate (6ca’)

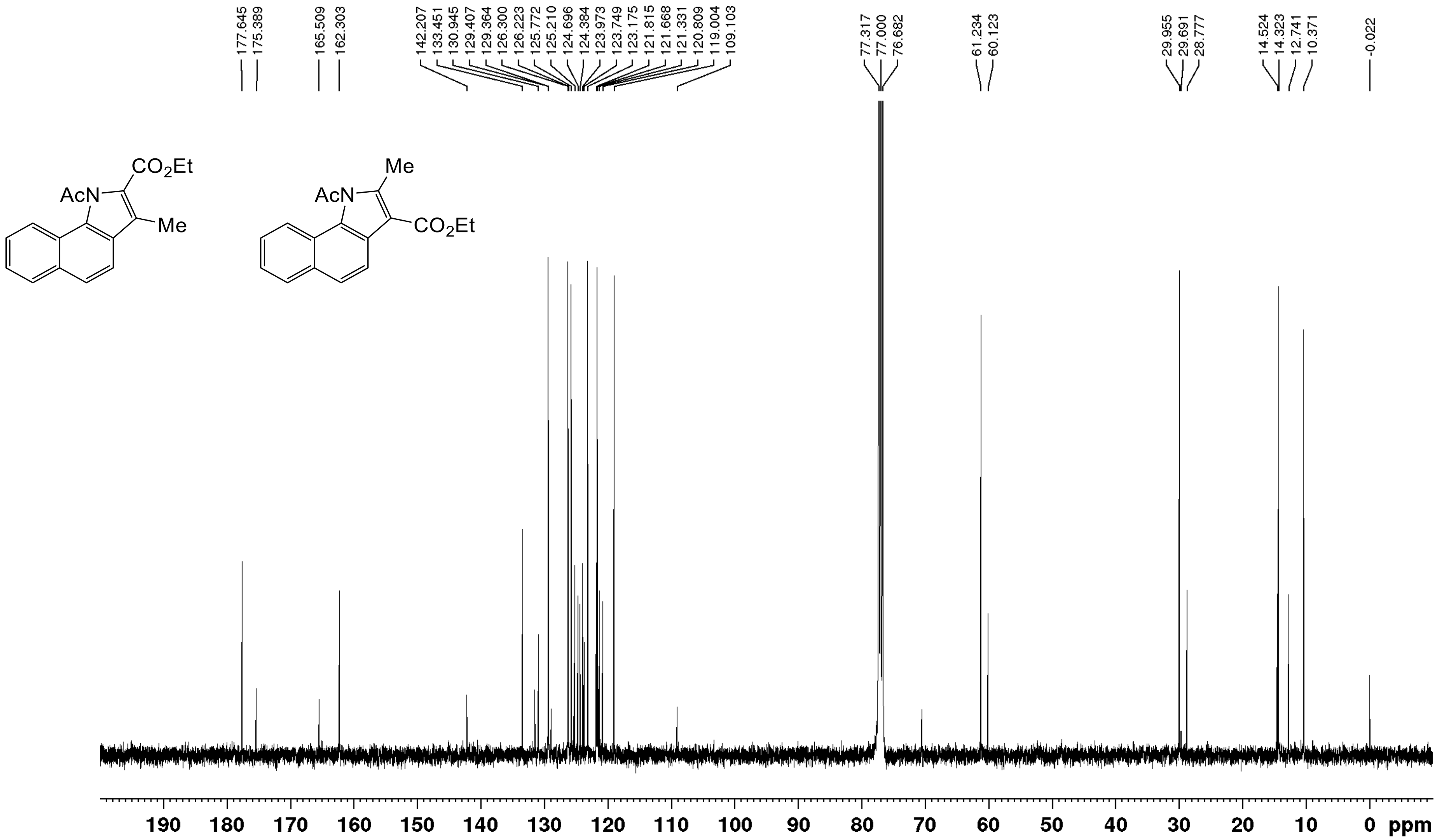

^{1}H NMR of a mixture of 5ca, 6ca, 6ca', and 7ca with C_6Me_6 in DMSO-d_6

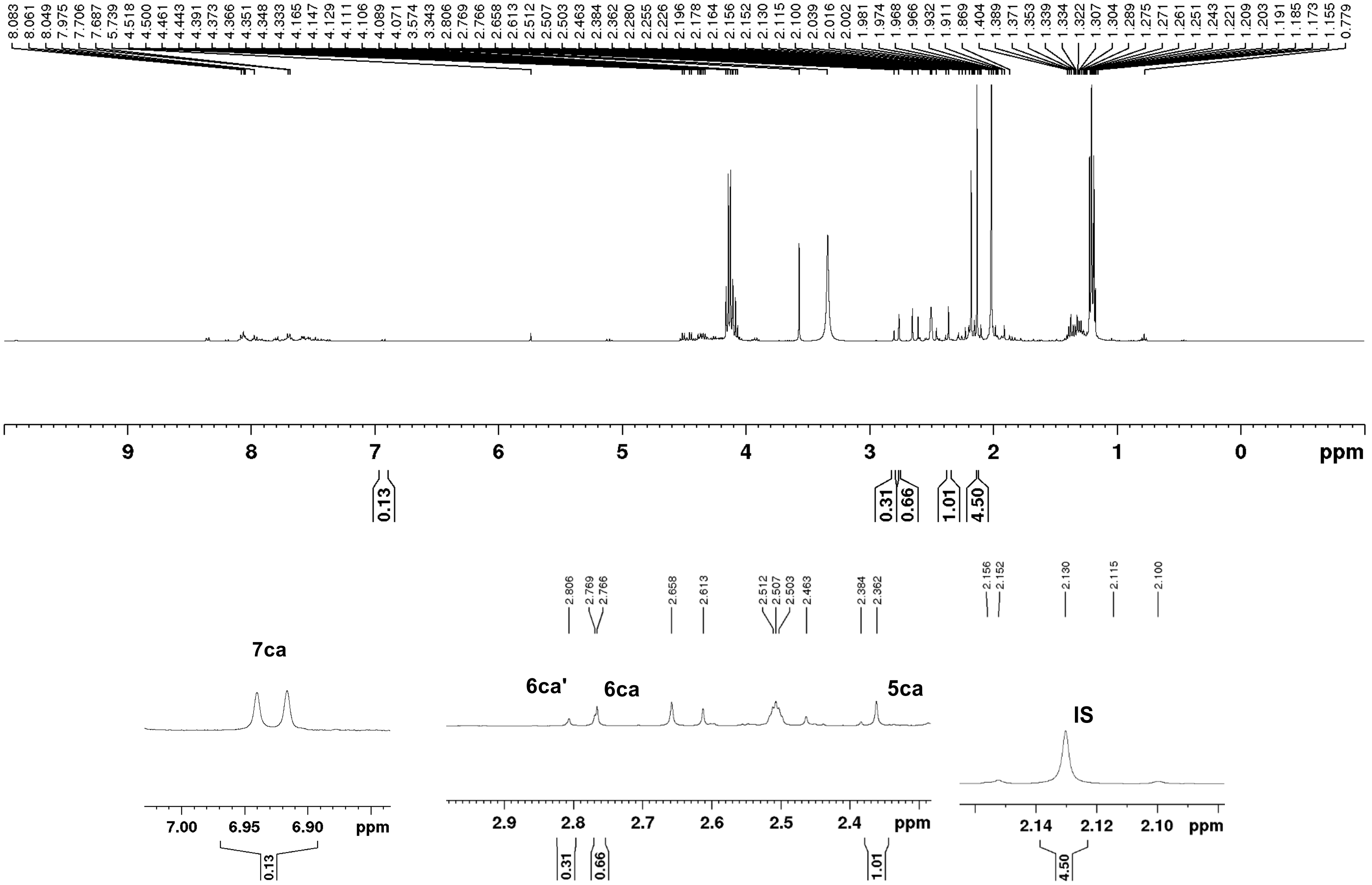

Dimethyl 1,3-dimethylphenanthrene-2,4-dicarboxylate (5cb)

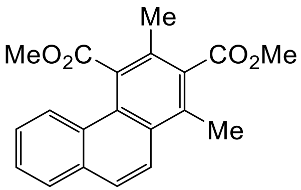

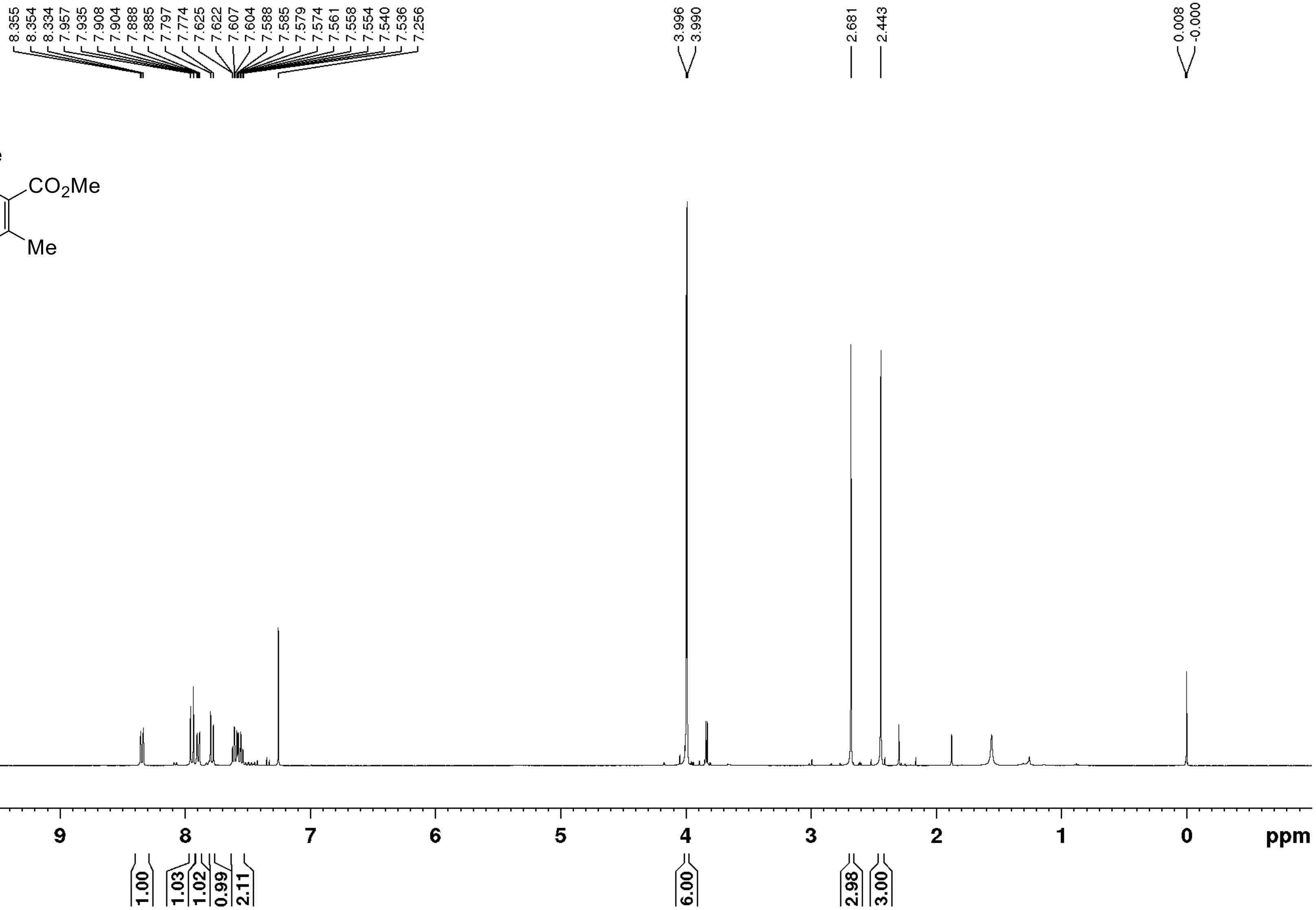

Dimethyl 1,3-dimethylphenanthrene-2,4-dicarboxylate (5cb)

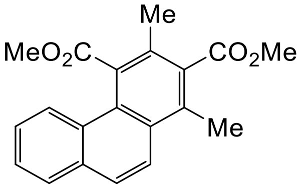

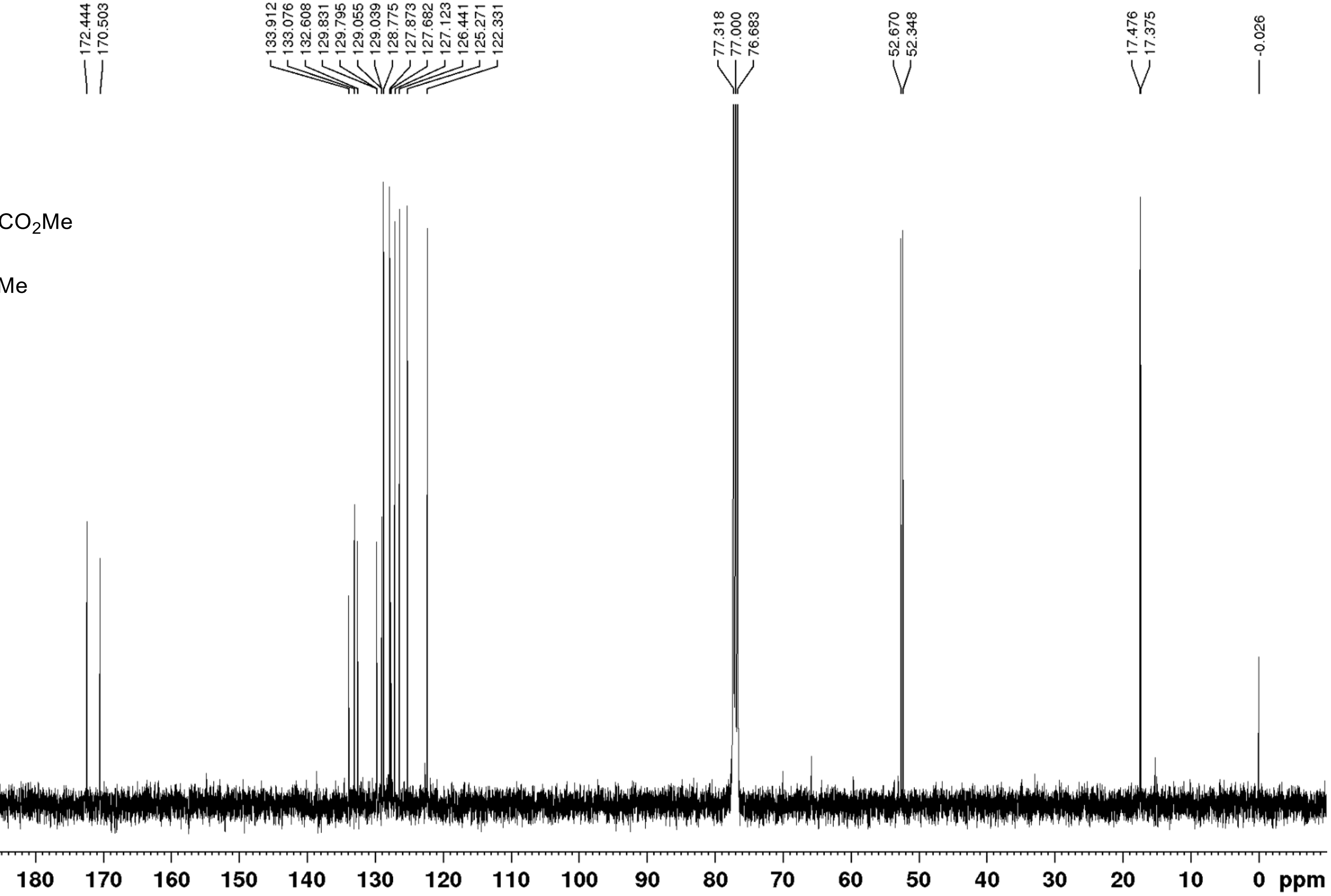

Methyl 1-acetyl-3-methyl-1*H*-benzo[*g*]indole-2-carboxylate (6cb) and methyl 1-acetyl-2-methyl-1*H*-benzo[*g*]indole-3-carboxylate (6cb')

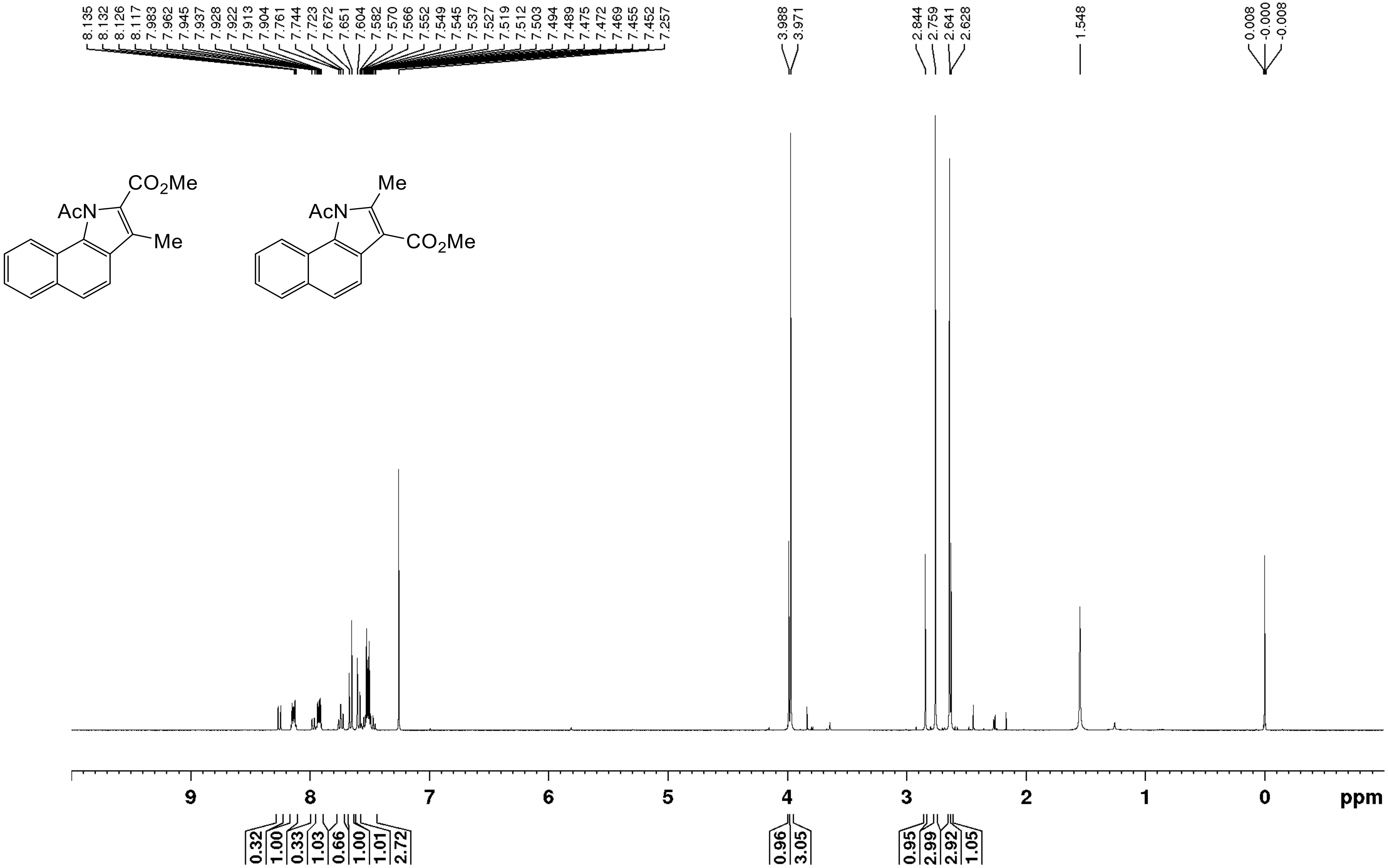

Methyl 1-acetyl-3-methyl-1*H*-benzo[*g*]indole-2-carboxylate (6cb) and methyl 1-acetyl-2-methyl-1*H*-benzo[*g*]indole-3-carboxylate (6cb')

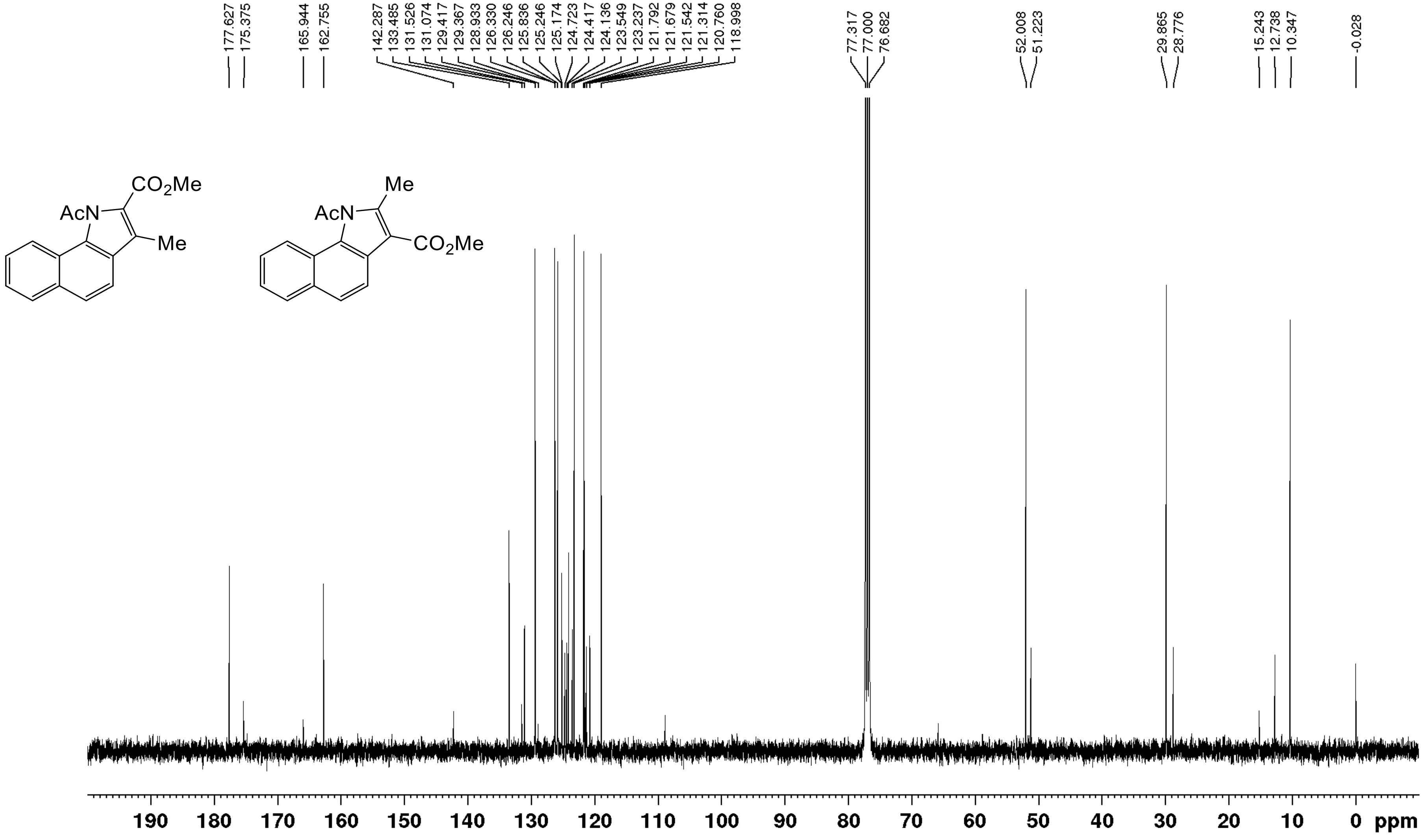

Diethyl 1,3-dibutylphenanthrene-2,4-dicarboxylate (5cc)

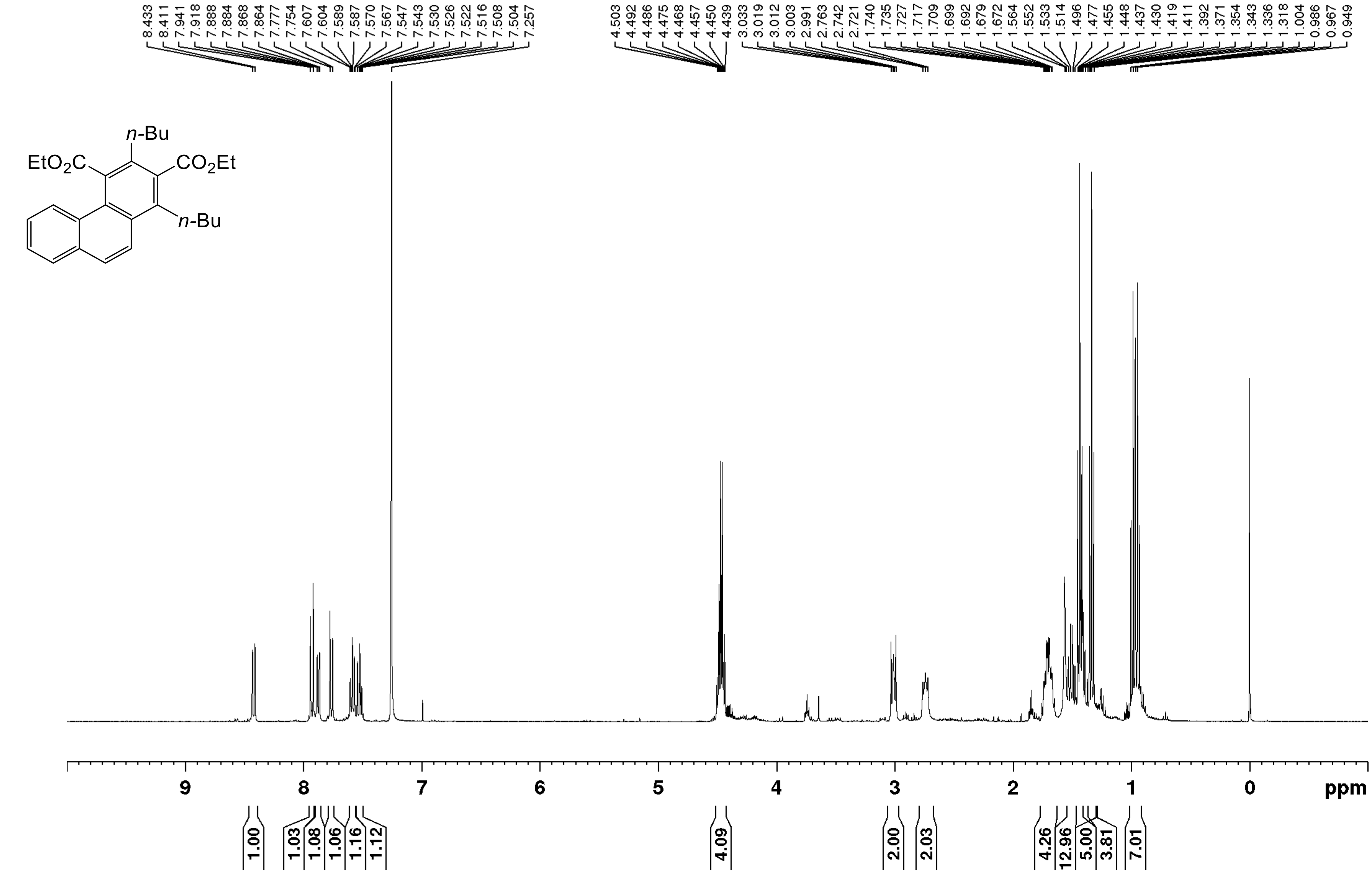

Diethyl 1,3-dibutylphenanthrene-2,4-dicarboxylate (5cc)

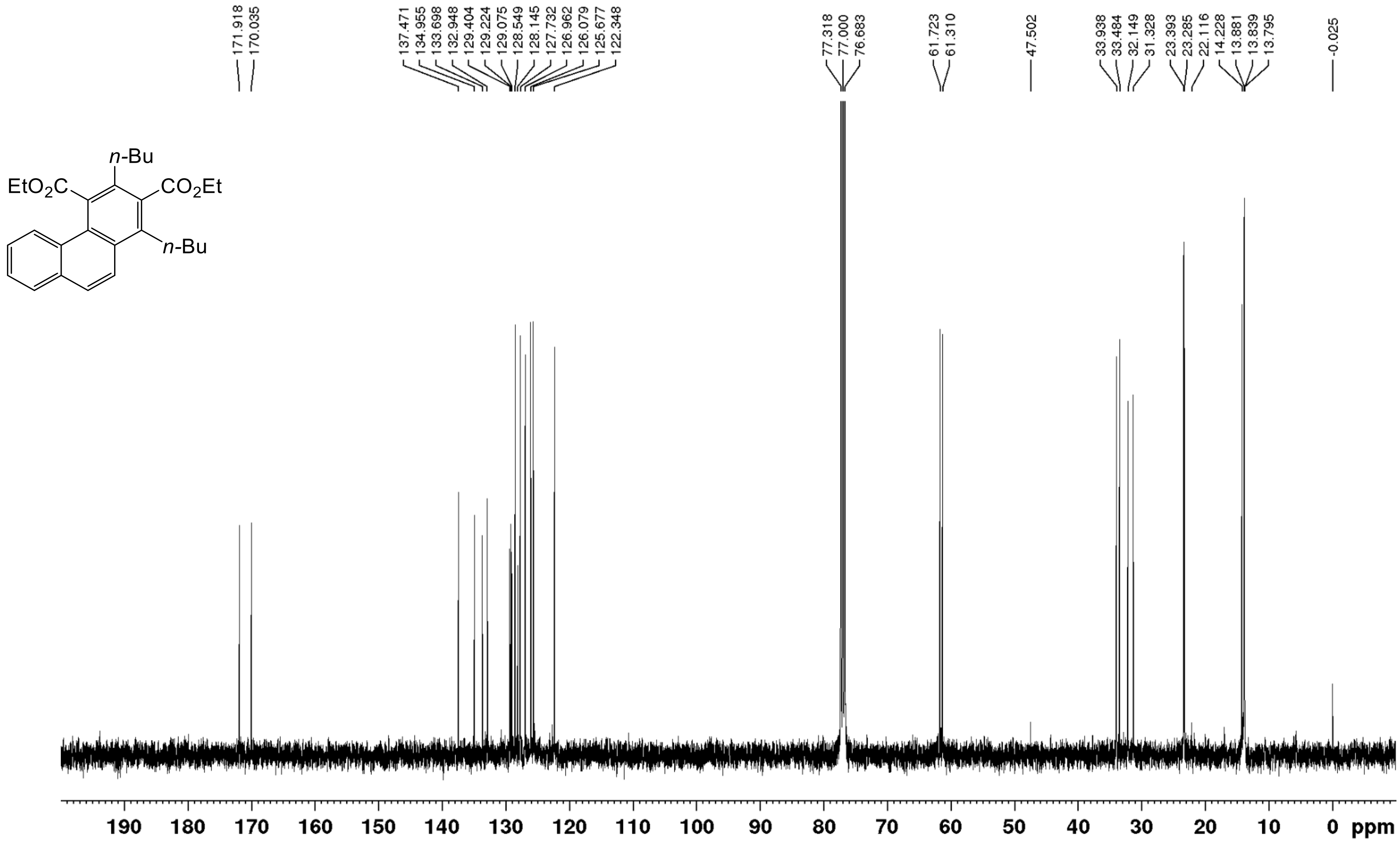

Diethyl 1,3-dibutylphenanthrene-2,4-dicarboxylate (5cc') and **diethyl 1,3-dibutylphenanthrene-2,4-dicarboxylate (6cc')**

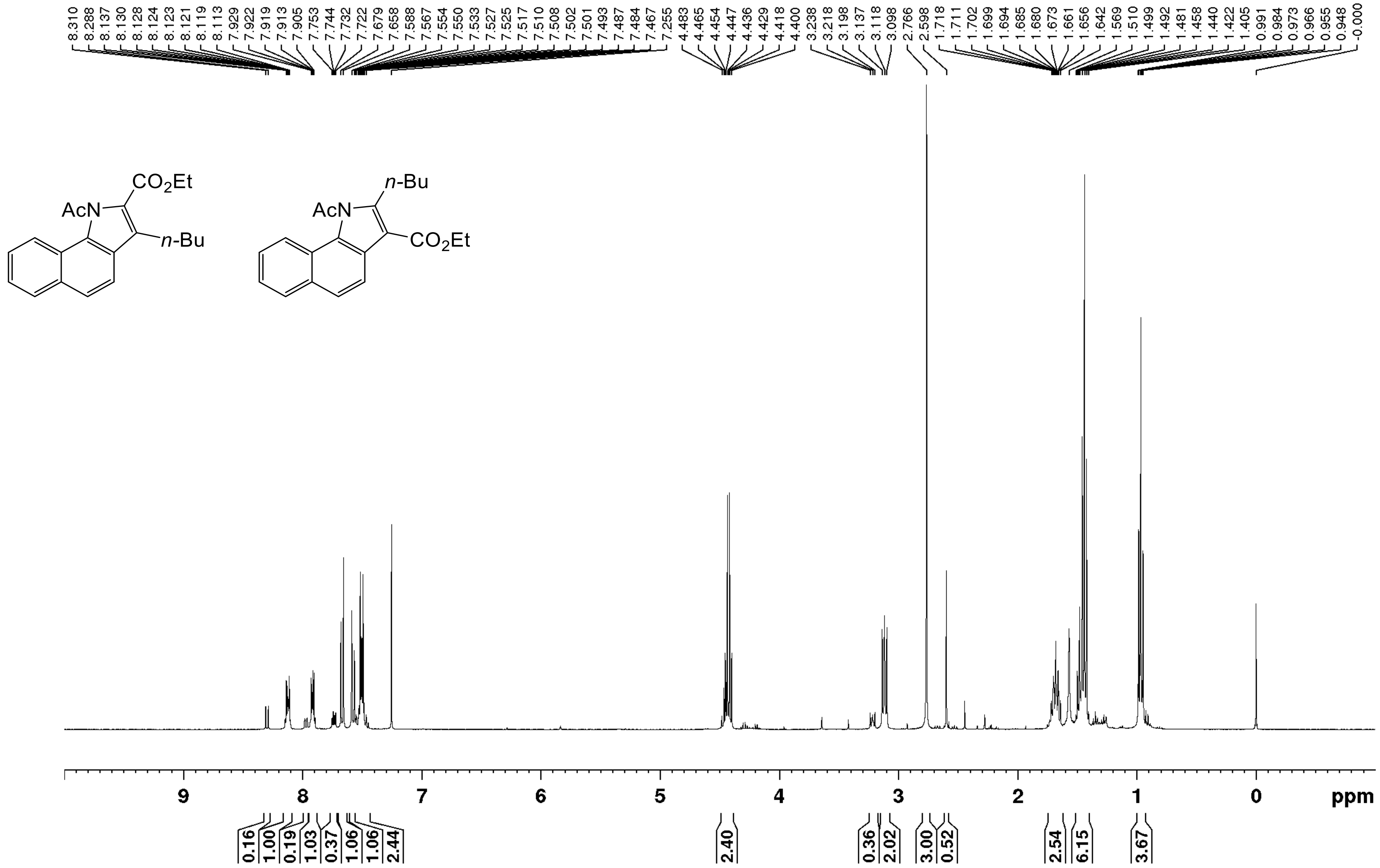

Ethyl 1-acetyl-3-butyl-1*H*-benzo[*g*]indole-2-carboxylate (6cc) and ethyl 1-acetyl-2-butyl-1*H*-benzo[*g*]indole-3-carboxylate (6cc')

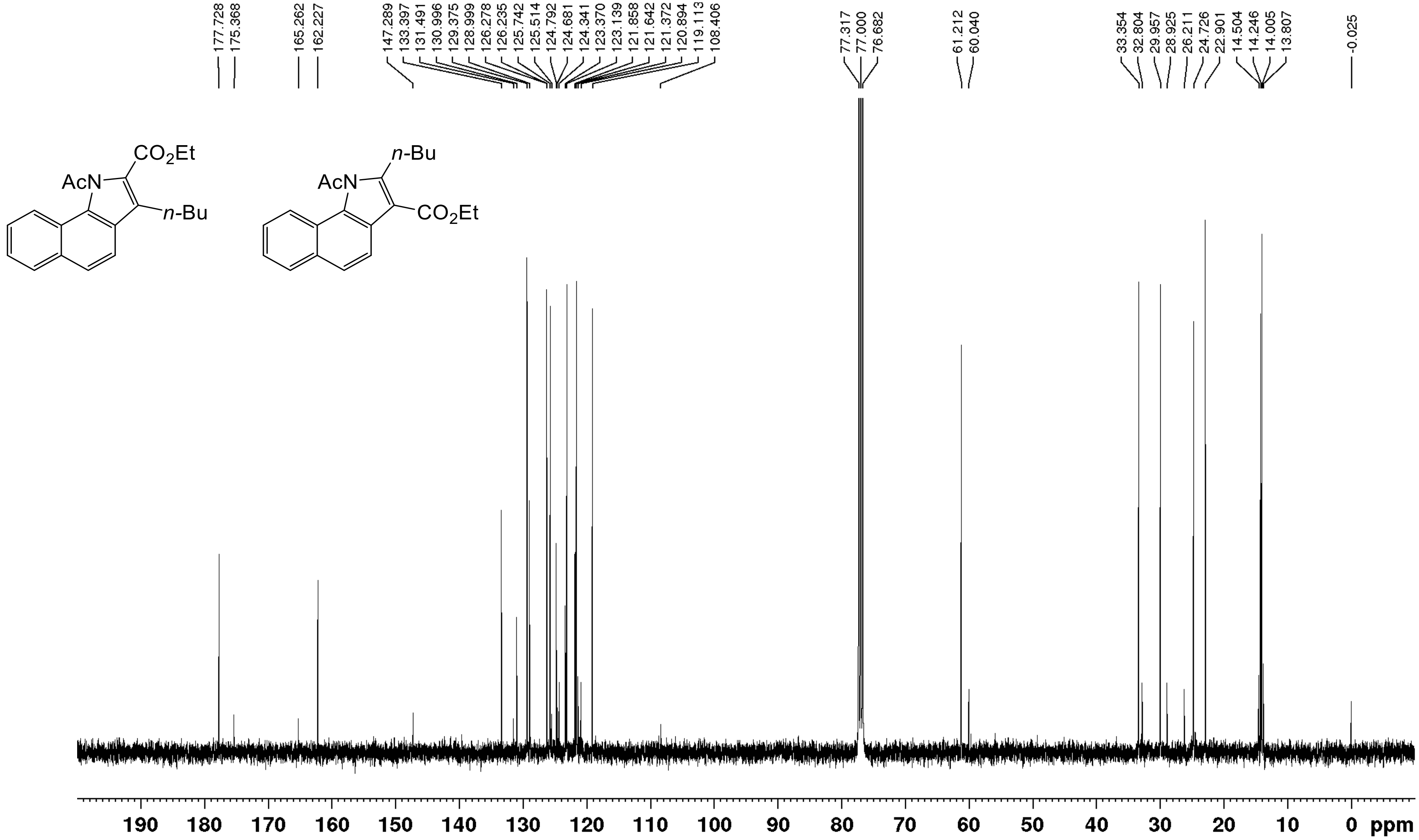

^{1}H NMR of a mixture of 5cc, 6cc, and 6cc' with C_6Me_6 in $CDCl_3$

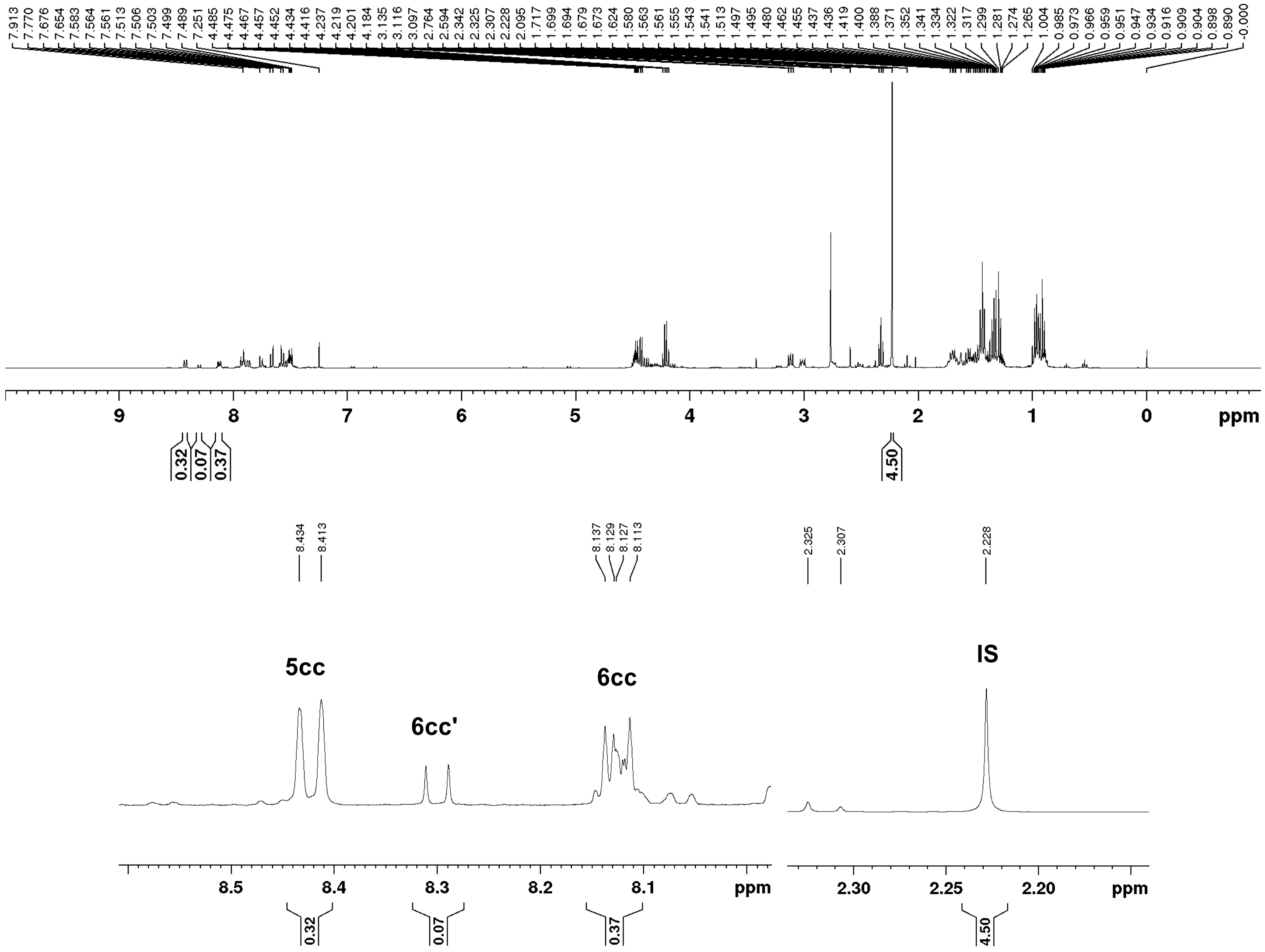

Dimethyl 1,3-bis(3-phenylpropyl)phenanthrene-2,4-dicarboxylate (5cd)

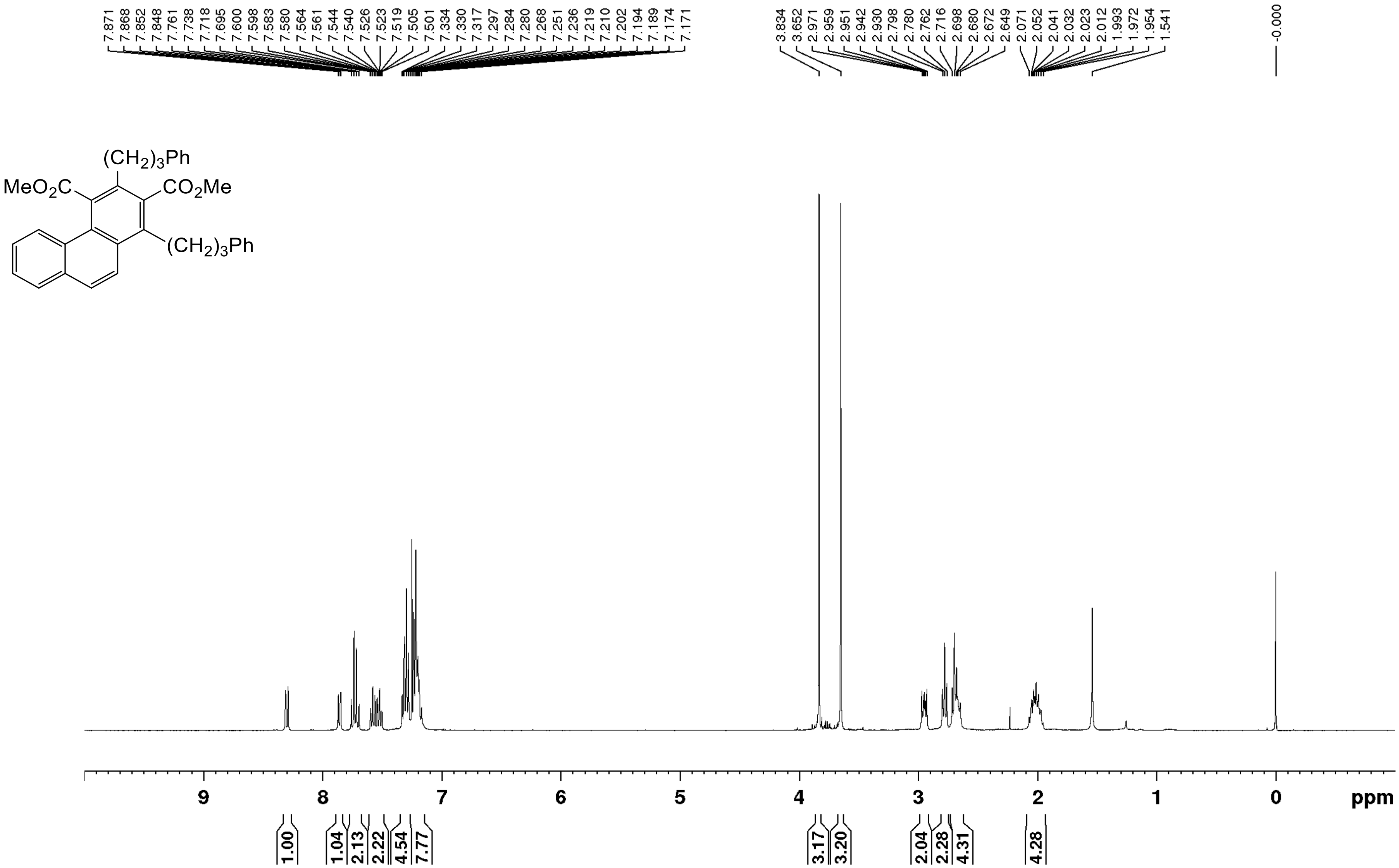

Dimethyl 1,3-bis(3-phenylpropyl)phenanthrene-2,4-dicarboxylate (5cd)

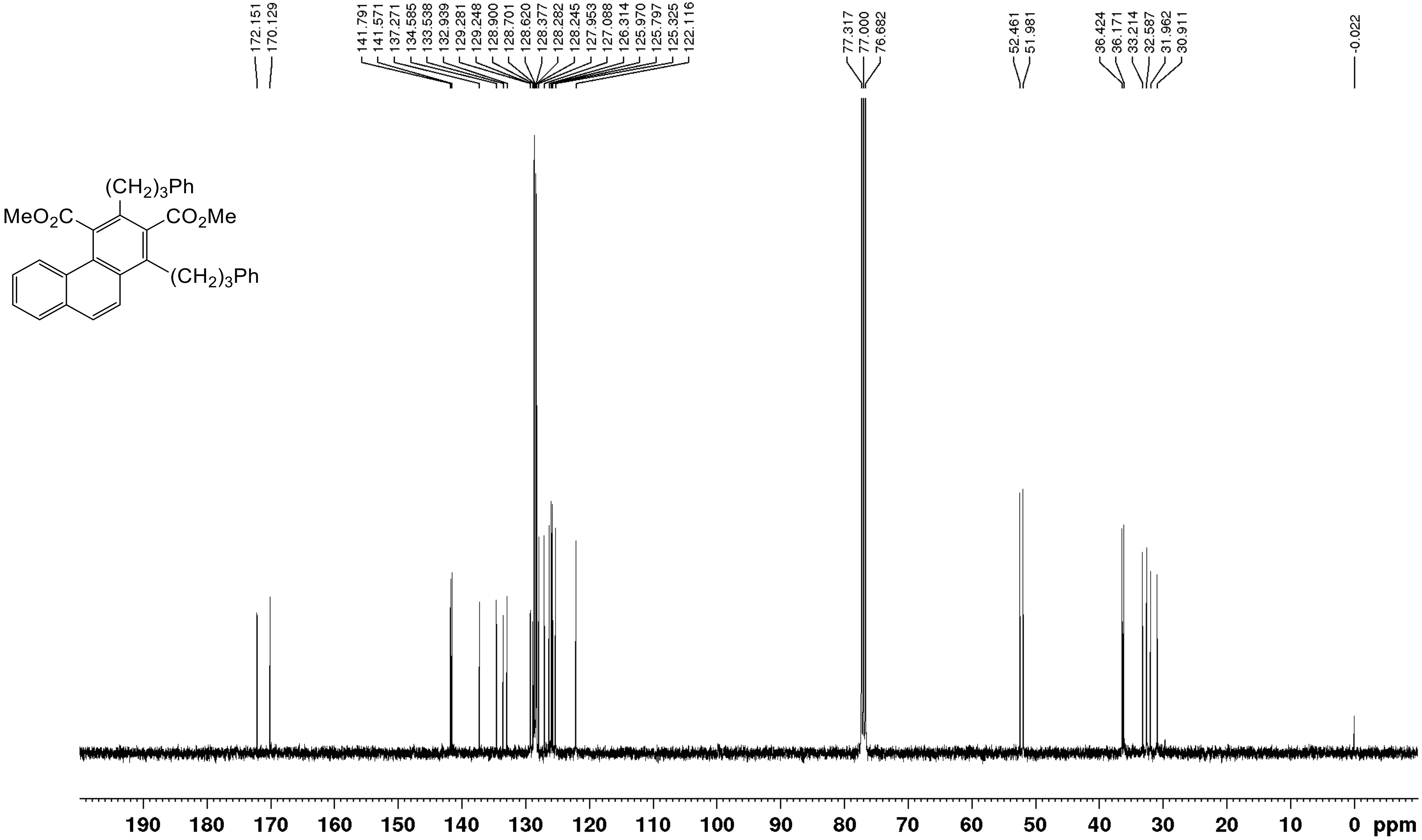

Methyl 1-acetyl-3-(3-phenylpropyl)-1*H*-benzo[*g*]indole-2-carboxylate (6cd) and methyl 1-acetyl-2-(3-phenylpropyl)-1*H*-benzo[*g*]indole-3-carboxylate (6cd')

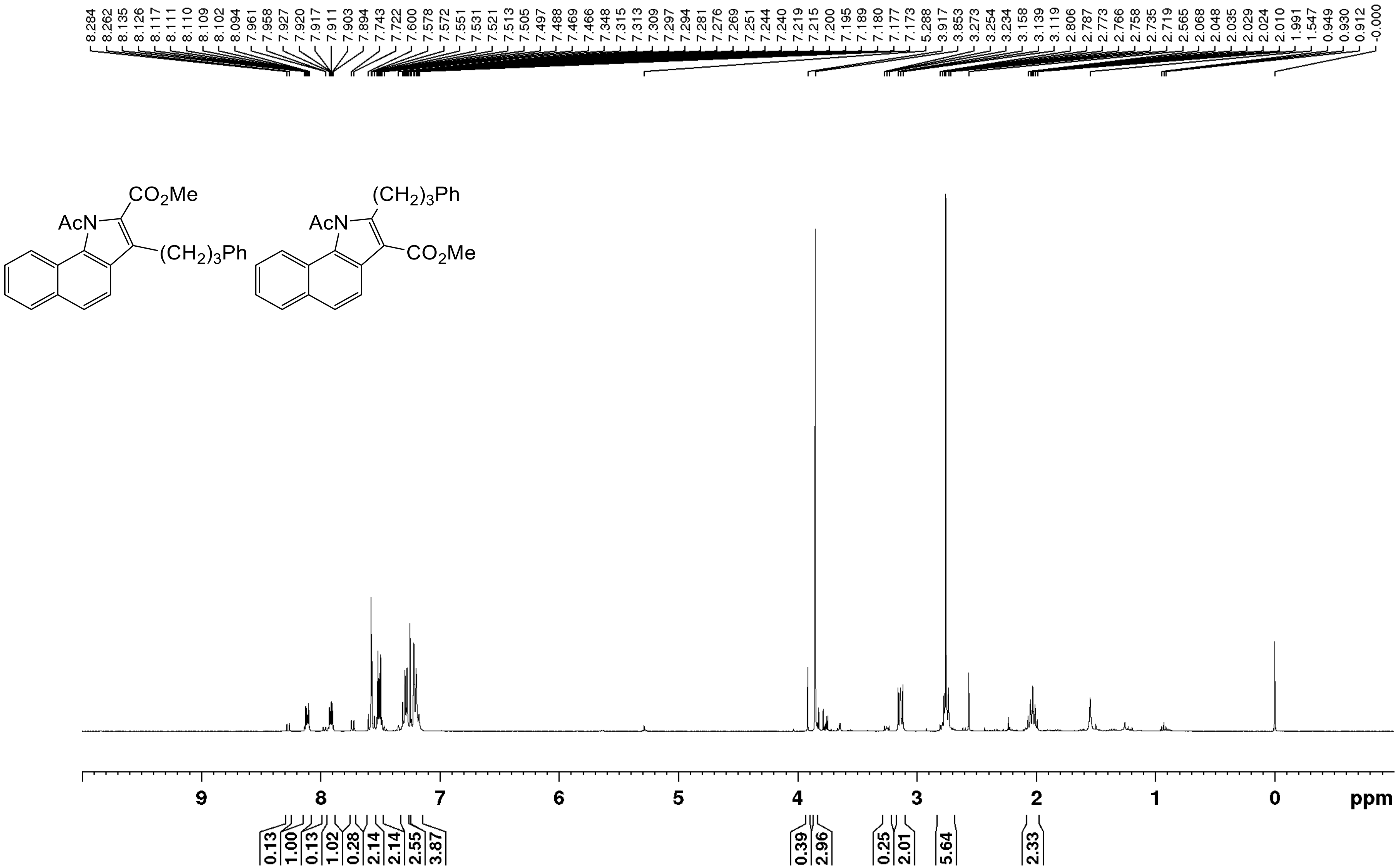

Methyl 1-acetyl-3-(3-phenylpropyl)-1*H*-benzo[*g*]indole-2-carboxylate (6cd) and methyl 1-acetyl-2-(3-phenylpropyl)-1*H*-benzo[*g*]indole-3-carboxylate (6cd')

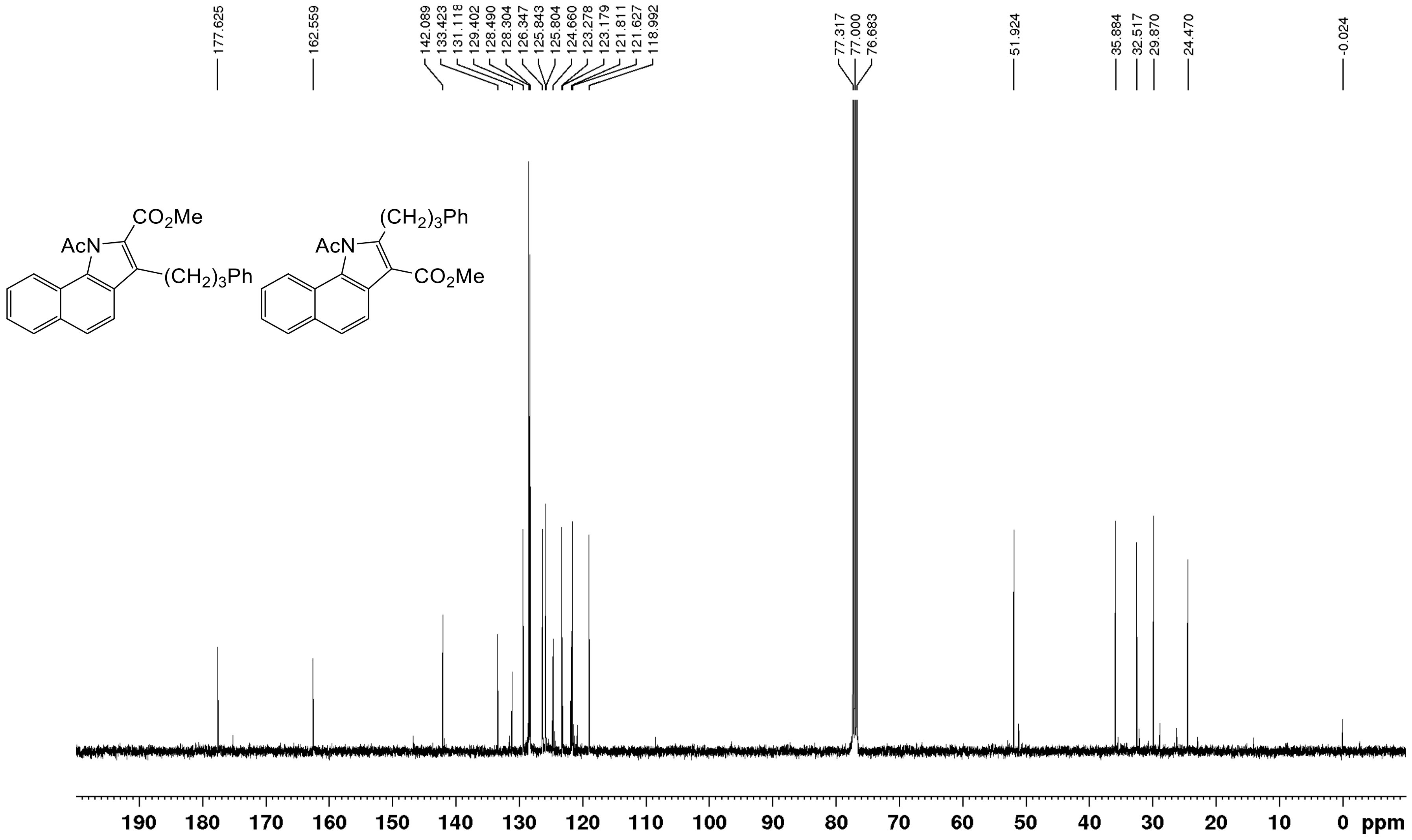

Dimethyl 1,3-bis(3-chloropropyl)phenanthrene-2,4-dicarboxylate (5ce)

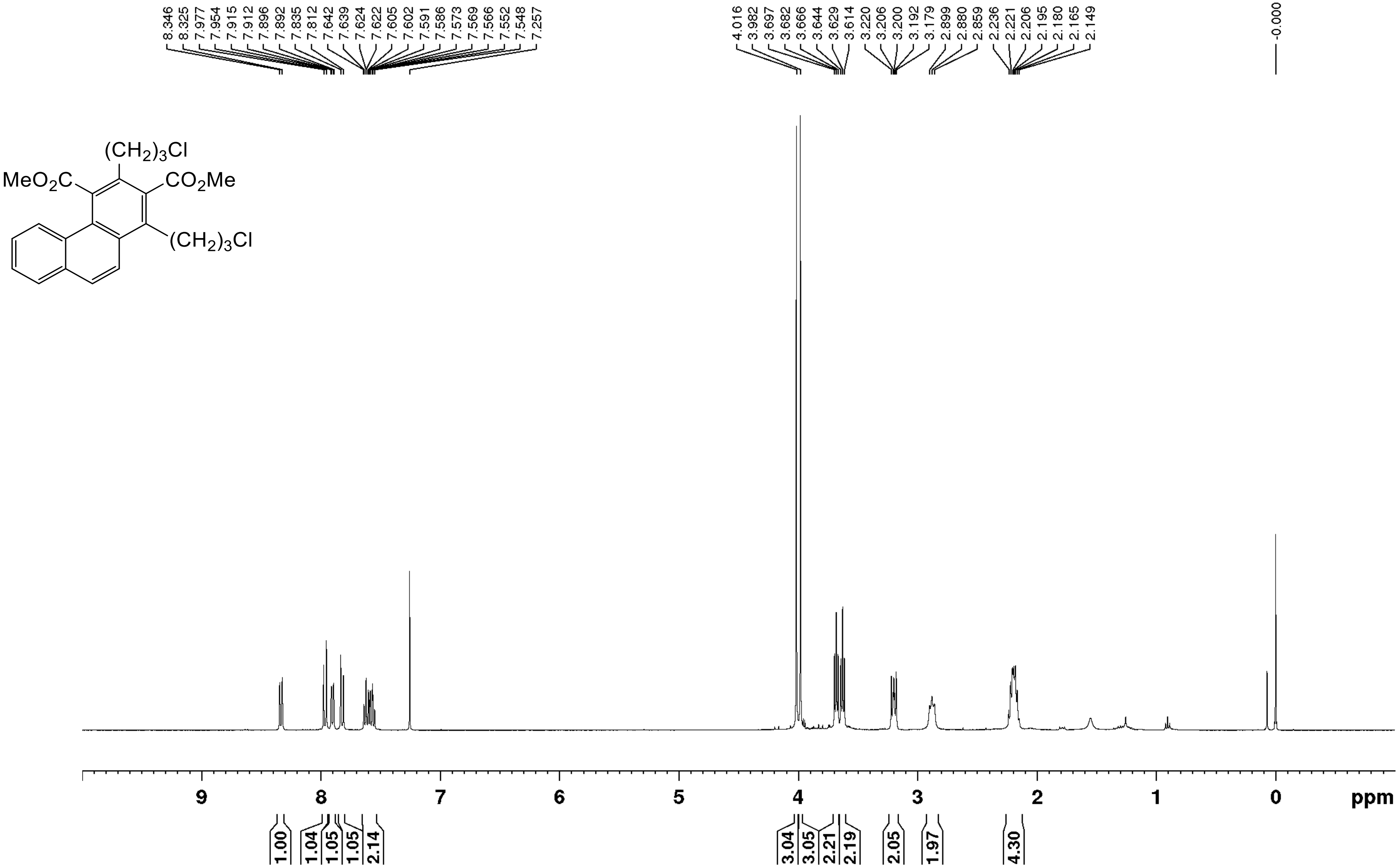

Dimethyl 1,3-bis(3-chloropropyl)phenanthrene-2,4-dicarboxylate (5ce)

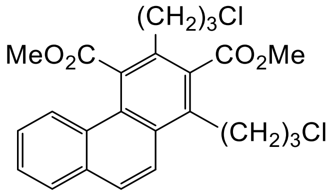

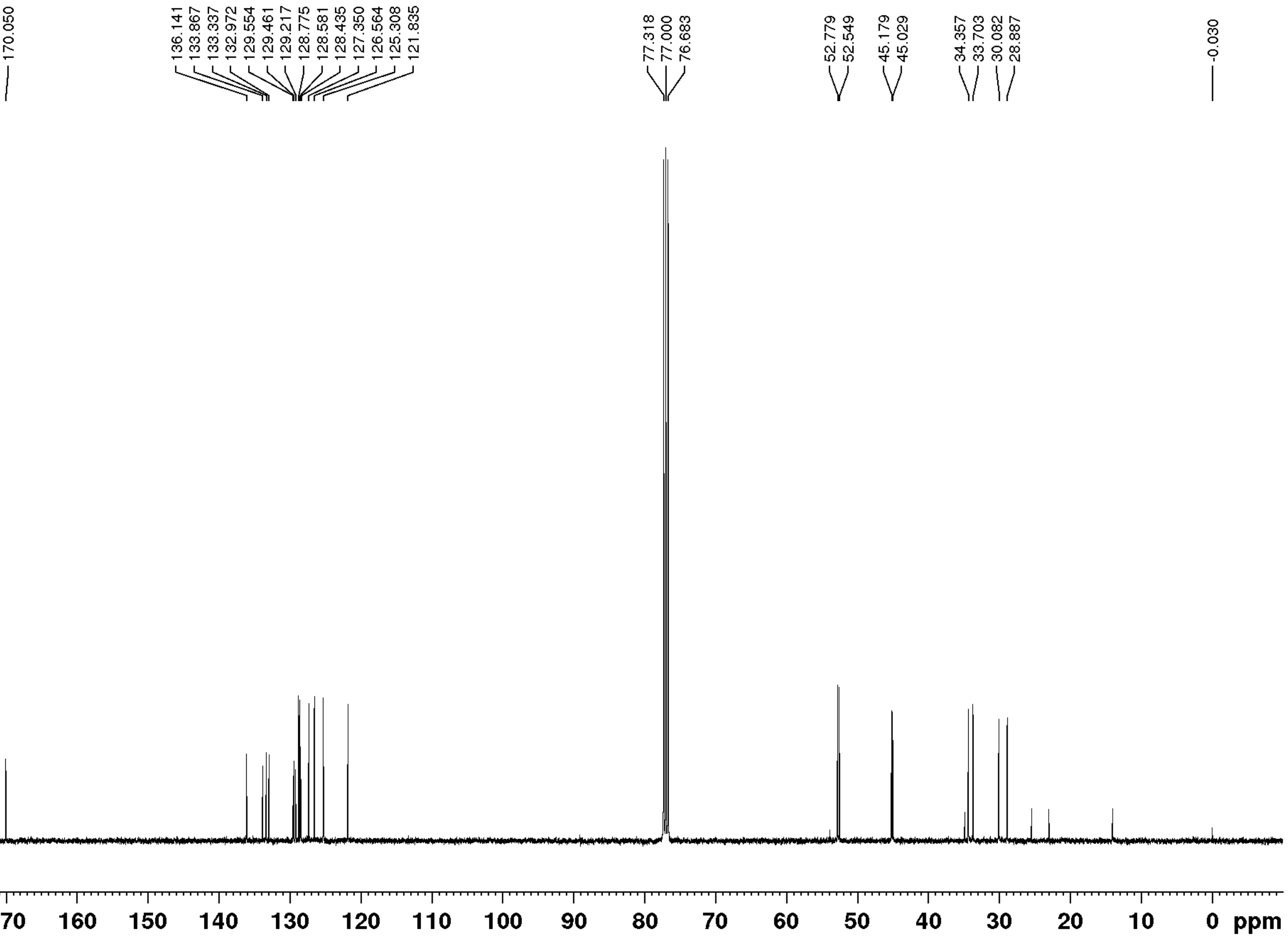

Methyl 1-acetyl-3-(3-chloropropyl)-1*H*-benzo[*g*]indole-2-carboxylate (6ce) and methyl 1-acetyl-2-(3-chloropropyl)-1*H*-benzo[*g*]indole-3-carboxylate (6ce')

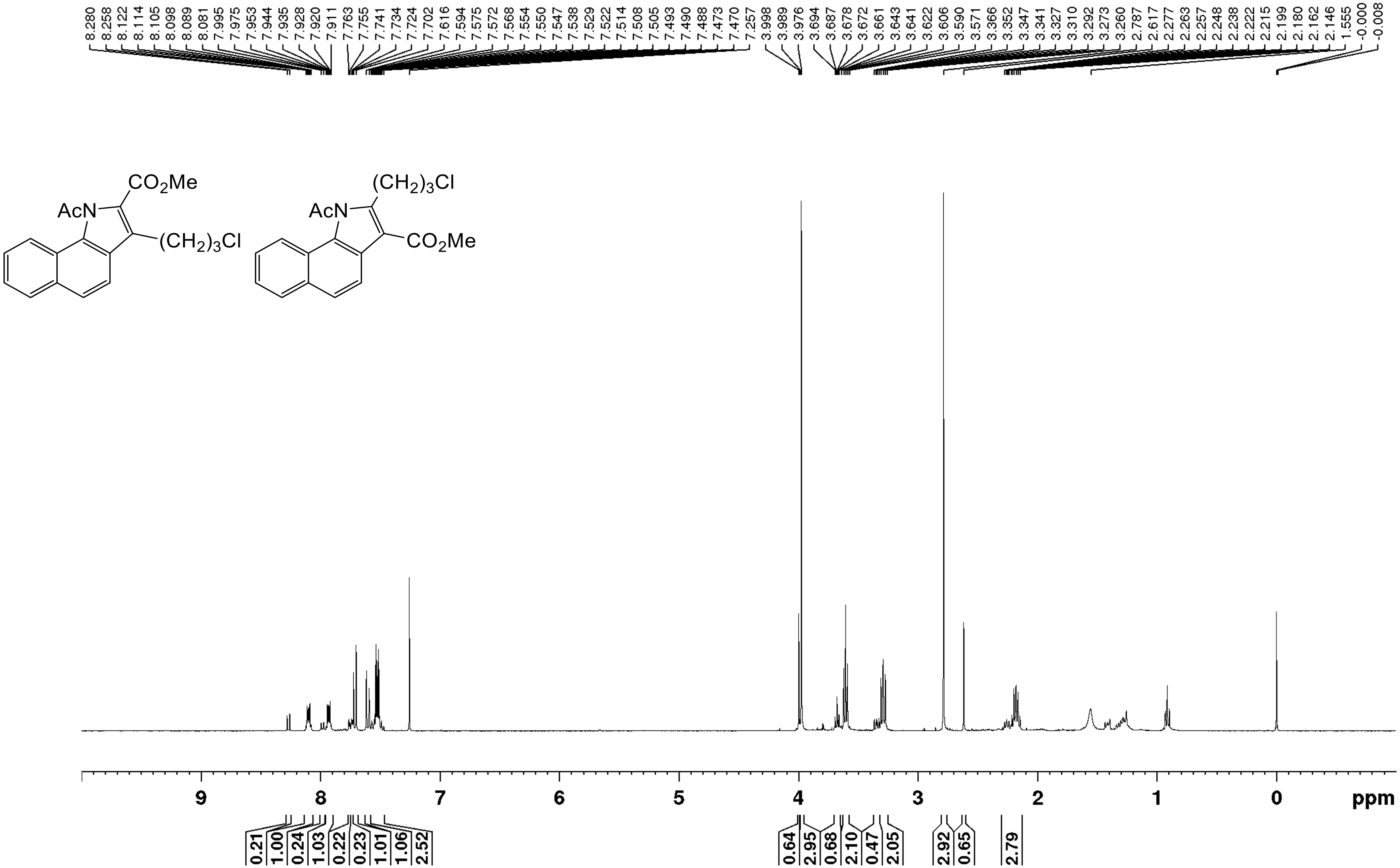

Methyl 1-acetyl-3-(3-chloropropyl)-1*H*-benzo[*g*]indole-2-carboxylate (6ce) and methyl 1-acetyl-2-(3-chloropropyl)-1*H*-benzo[*g*]indole-3-carboxylate (6ce')

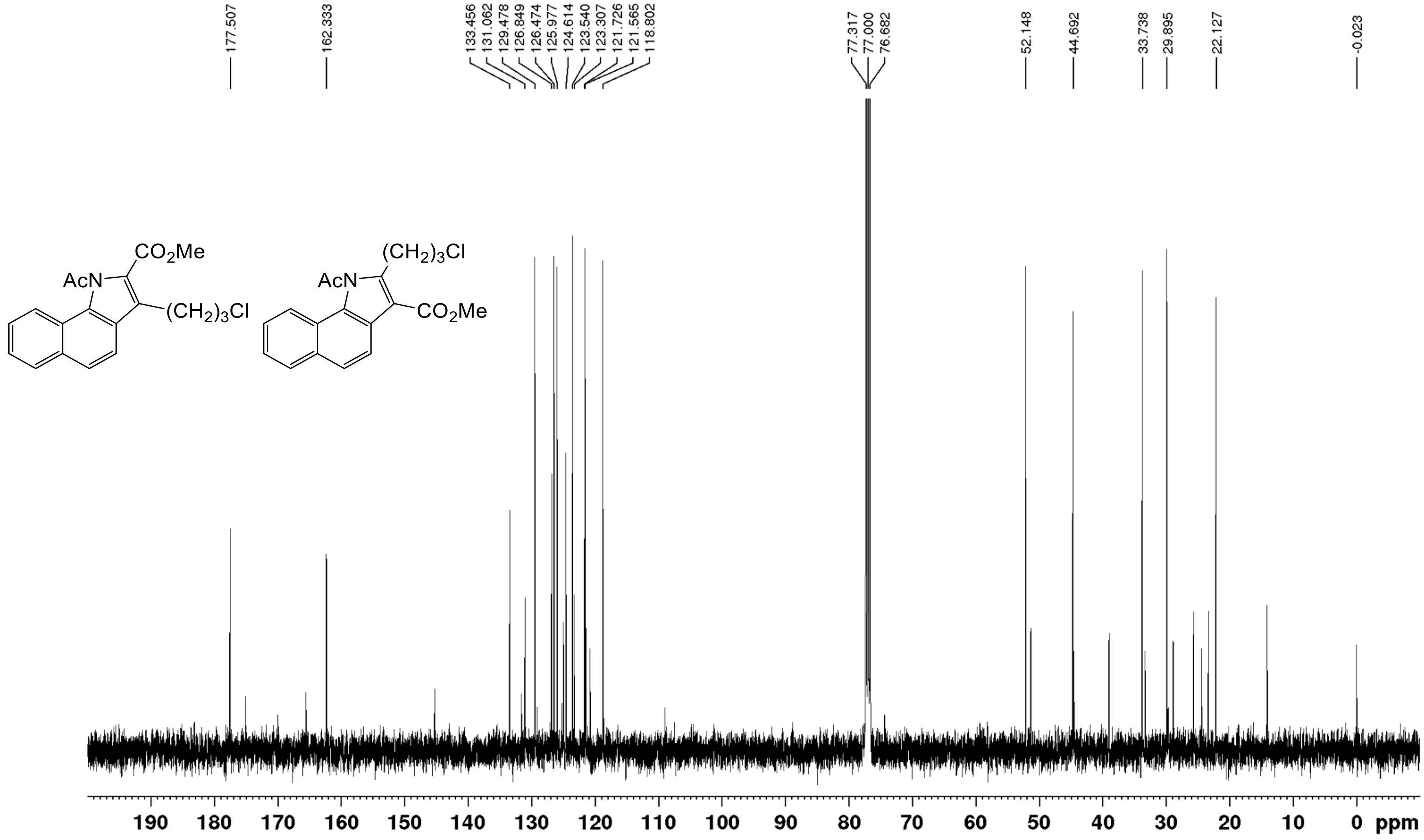

Ethyl 1-acetyl-2-phenyl-1*H*-benzo[*g*]indole-3-carboxylate (6cf')

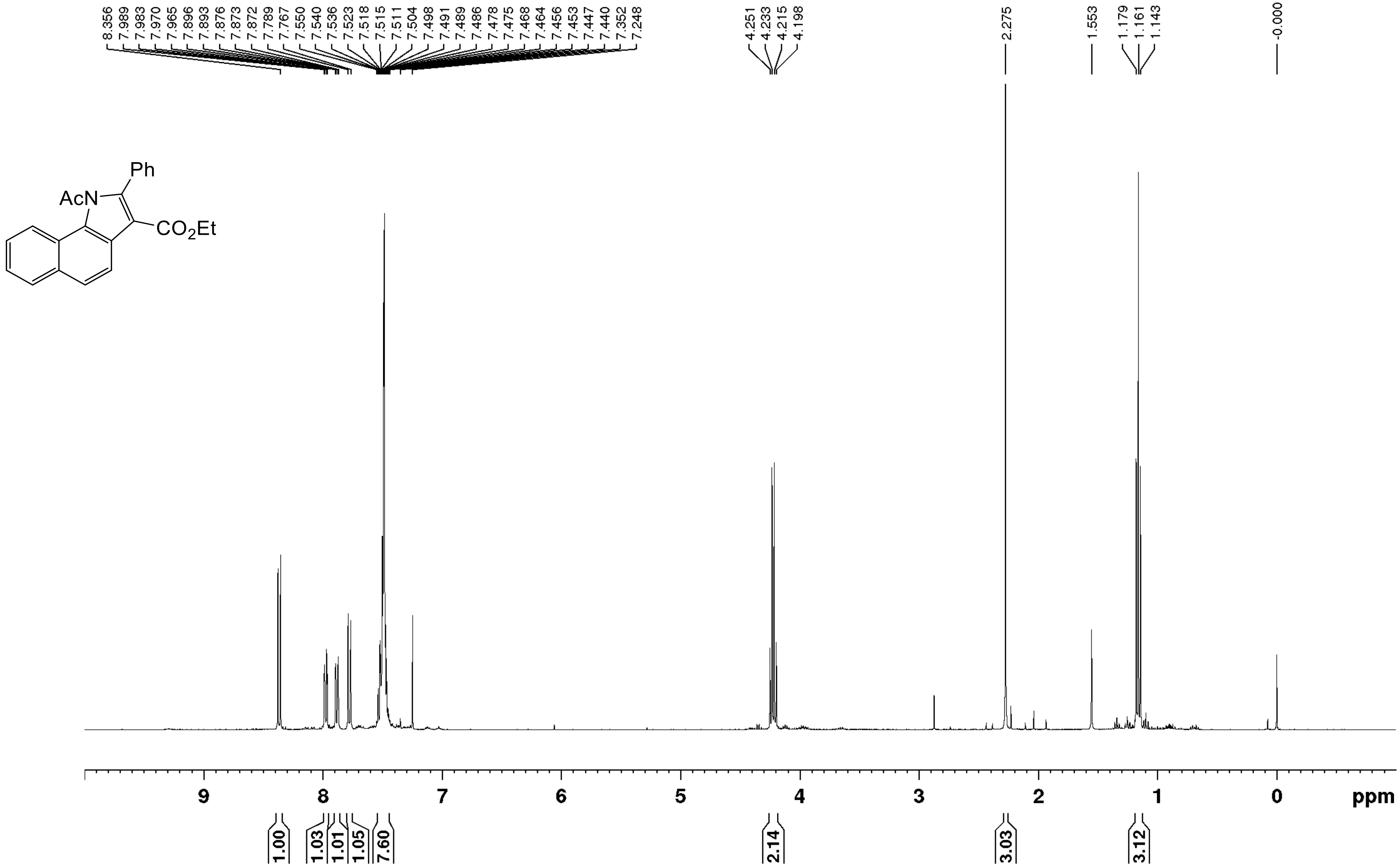

Ethyl 1-acetyl-2-phenyl-1*H*-benzo[*g*]indole-3-carboxylate (6cf')

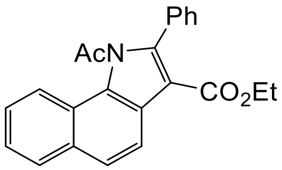

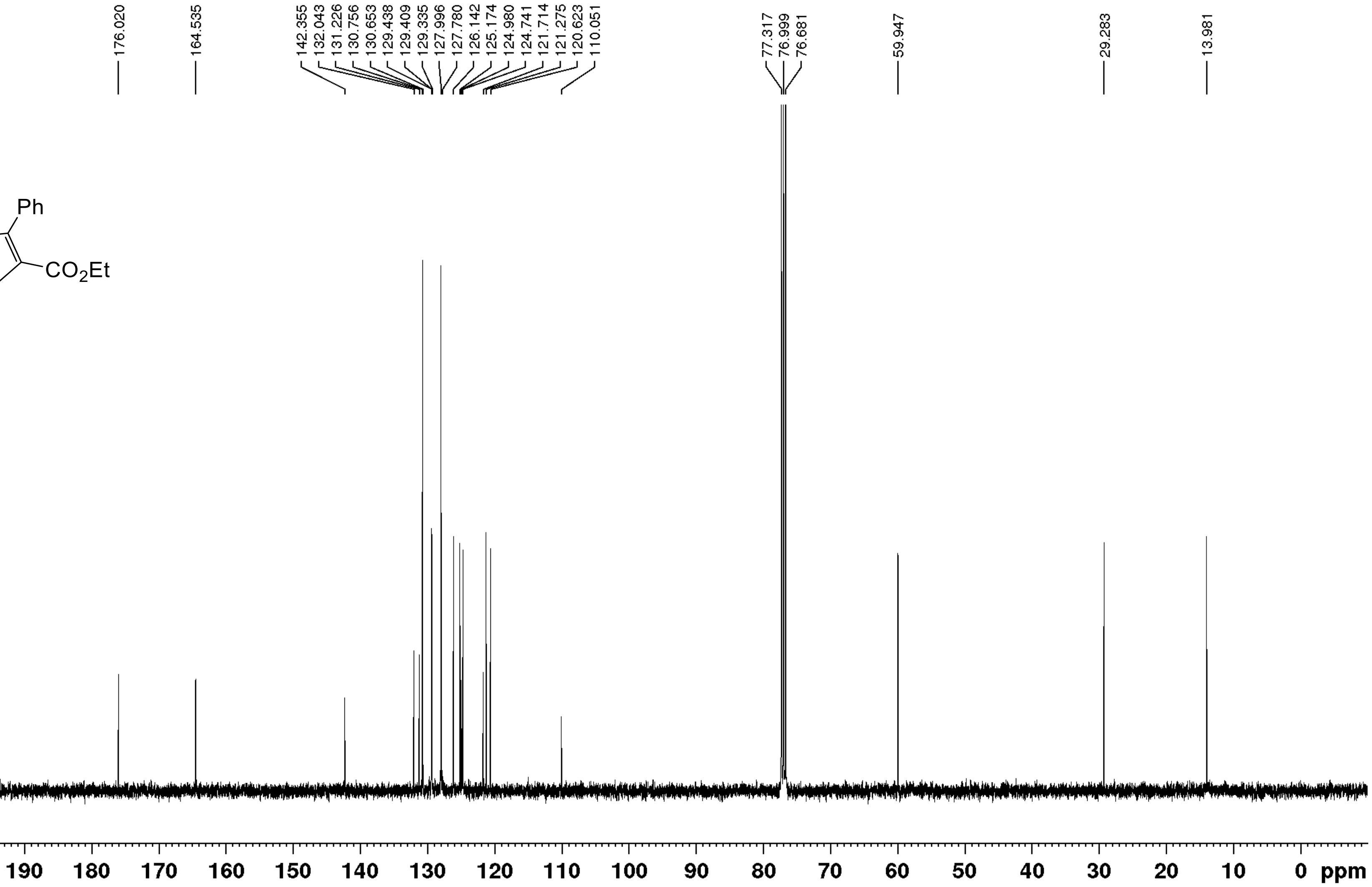

Ethyl 3-methyl-1-pivaloyl-1*H*-benzo[*g*]indole-2-carboxylate (6ea)

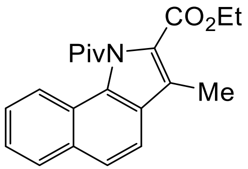

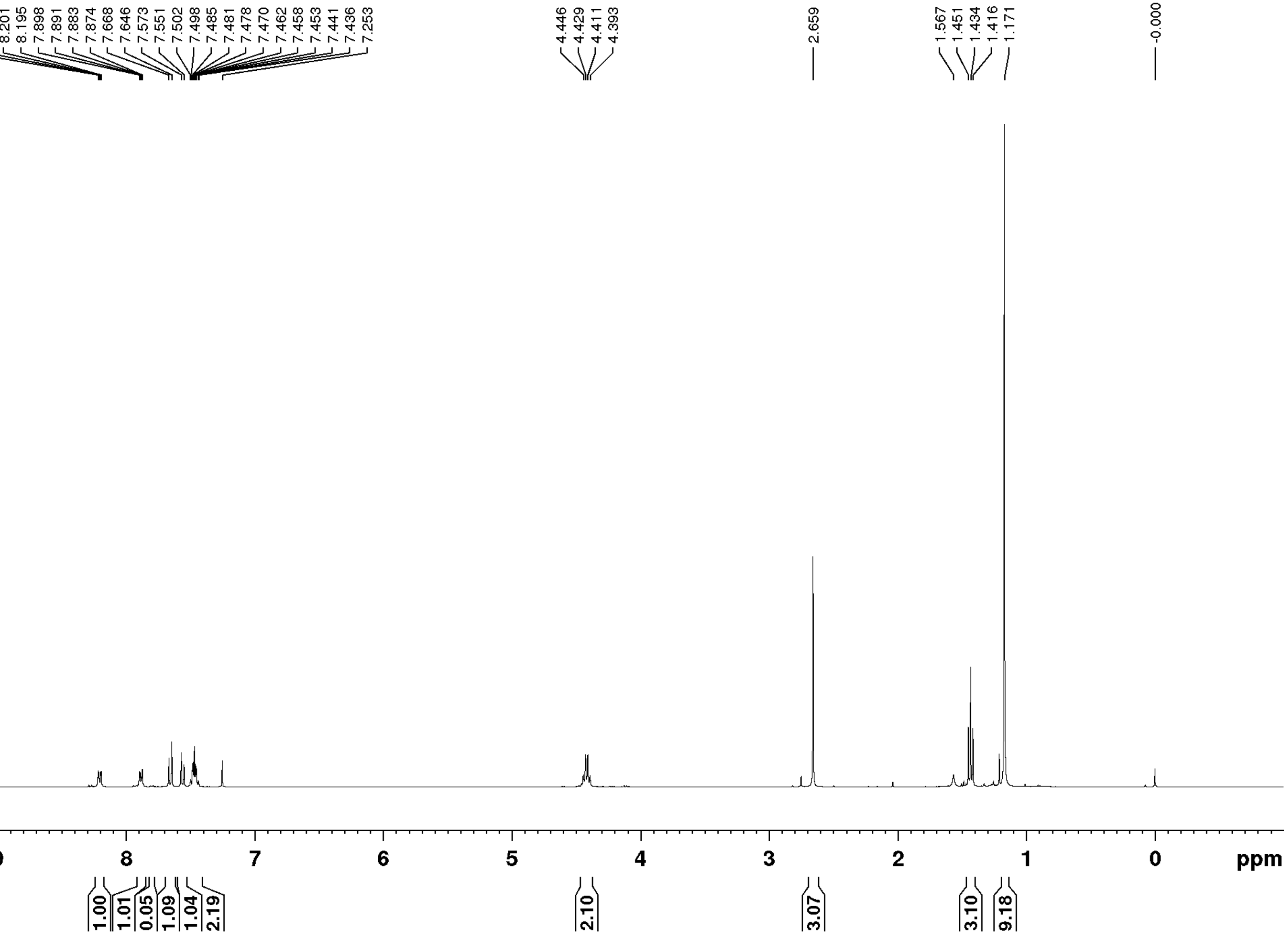

Ethyl 3-methyl-1-pivaloyl-1*H*-benzo[*g*]indole-2-carboxylate (6ea)

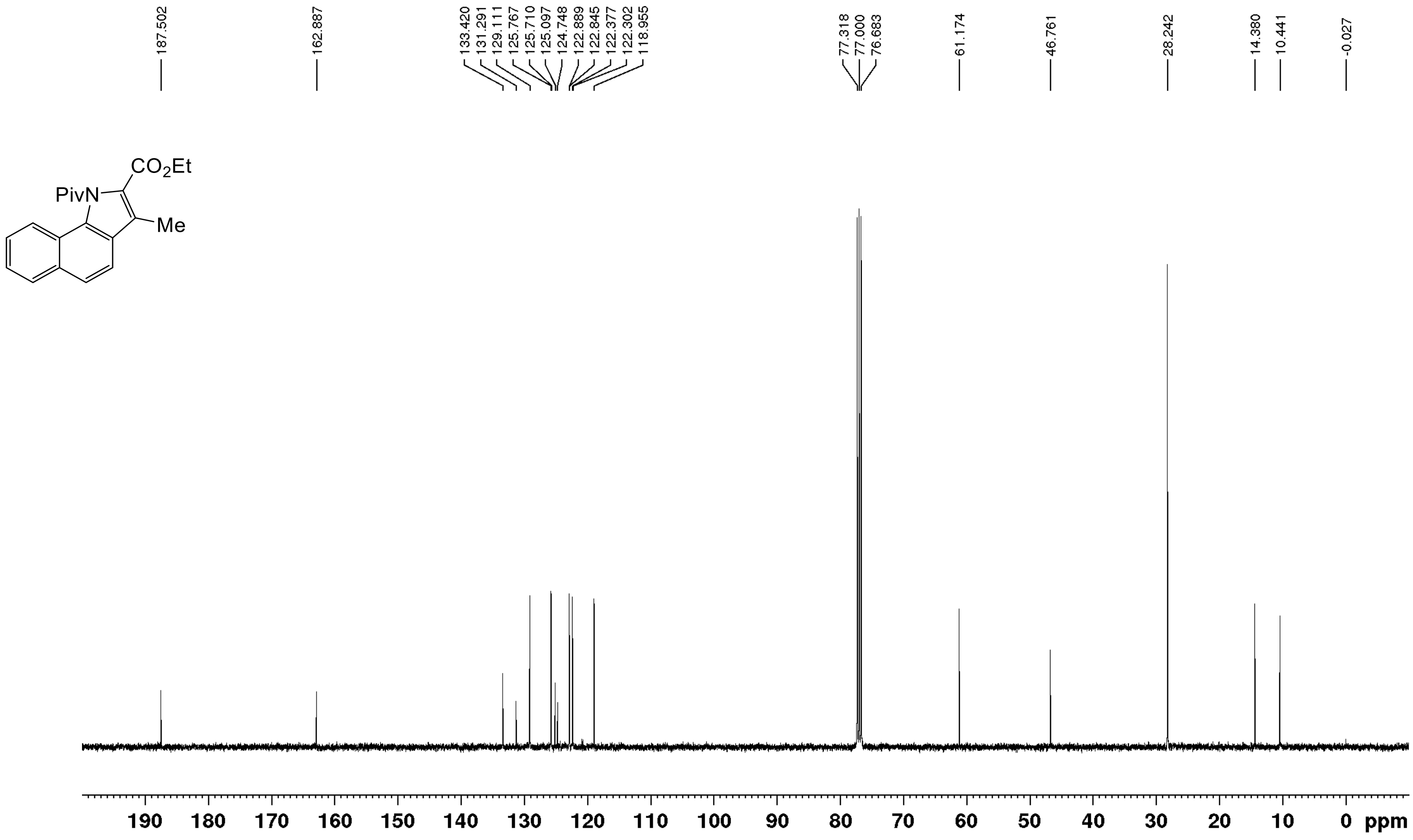

Diethyl (*Z*)-1'-(acetylimino)-3,5-dimethyl-1'*H*-spiro[cyclopentane-1,2'-naphthalene]-2,4-diene-2,4-dicarboxylate (7ca)

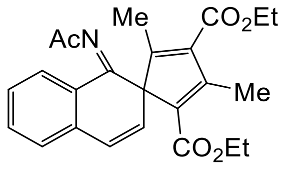

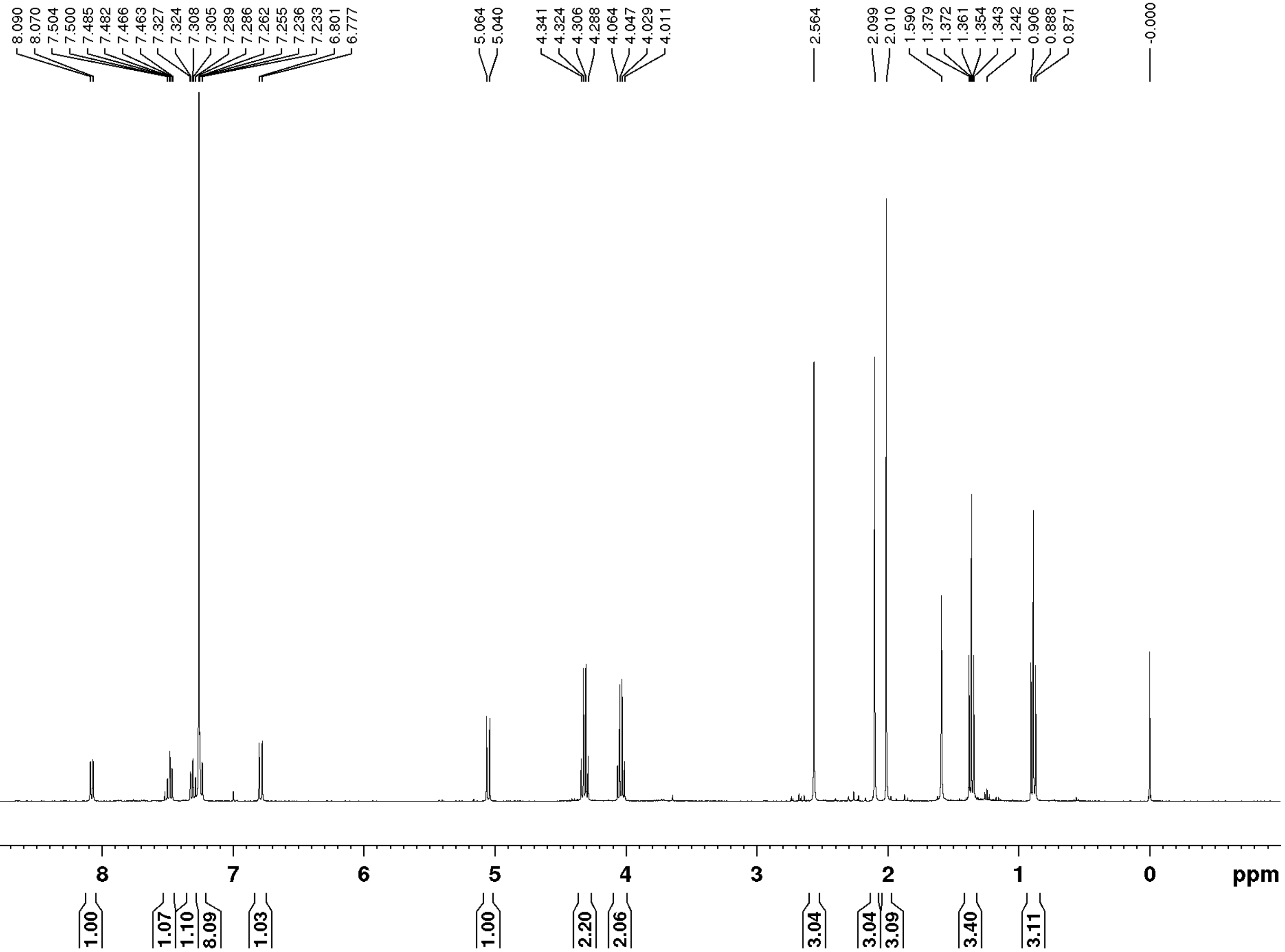

Diethyl (*Z*)-1'-(acetylimino)-3,5-dimethyl-1'*H*-spiro[cyclopentane-1,2'-naphthalene]-2,4-diene-2,4-dicarboxylate (7ca)

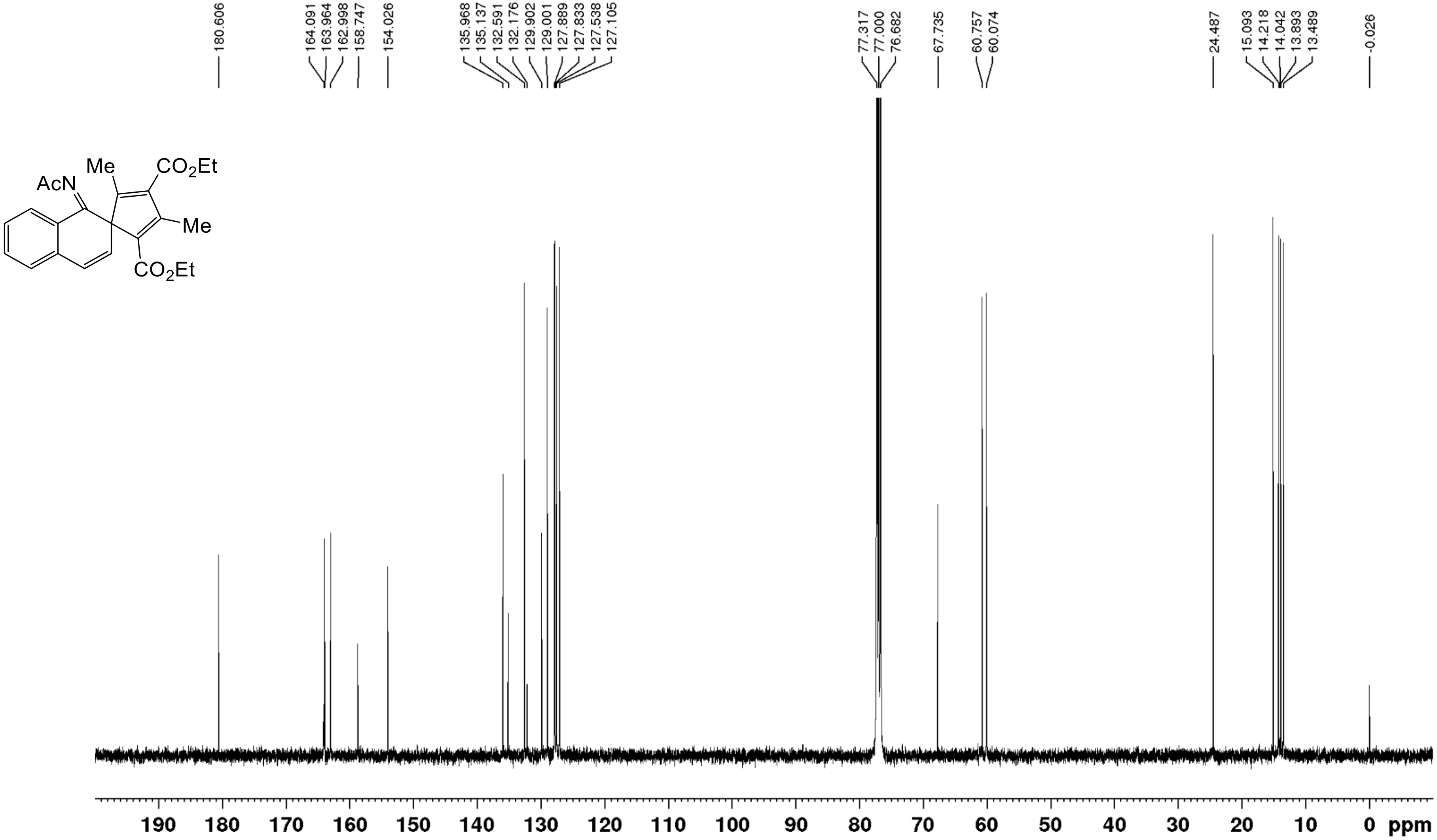

Diethyl (*Z*)-1'-(acetylimino)-3,5-dimethyl-1'*H*-spiro[cyclopentane-1,2'-naphthalene]-2,4-diene-2,4-dicarboxylate (7ca) (HSQC)

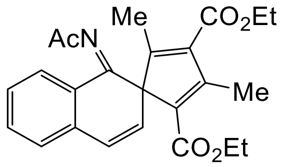

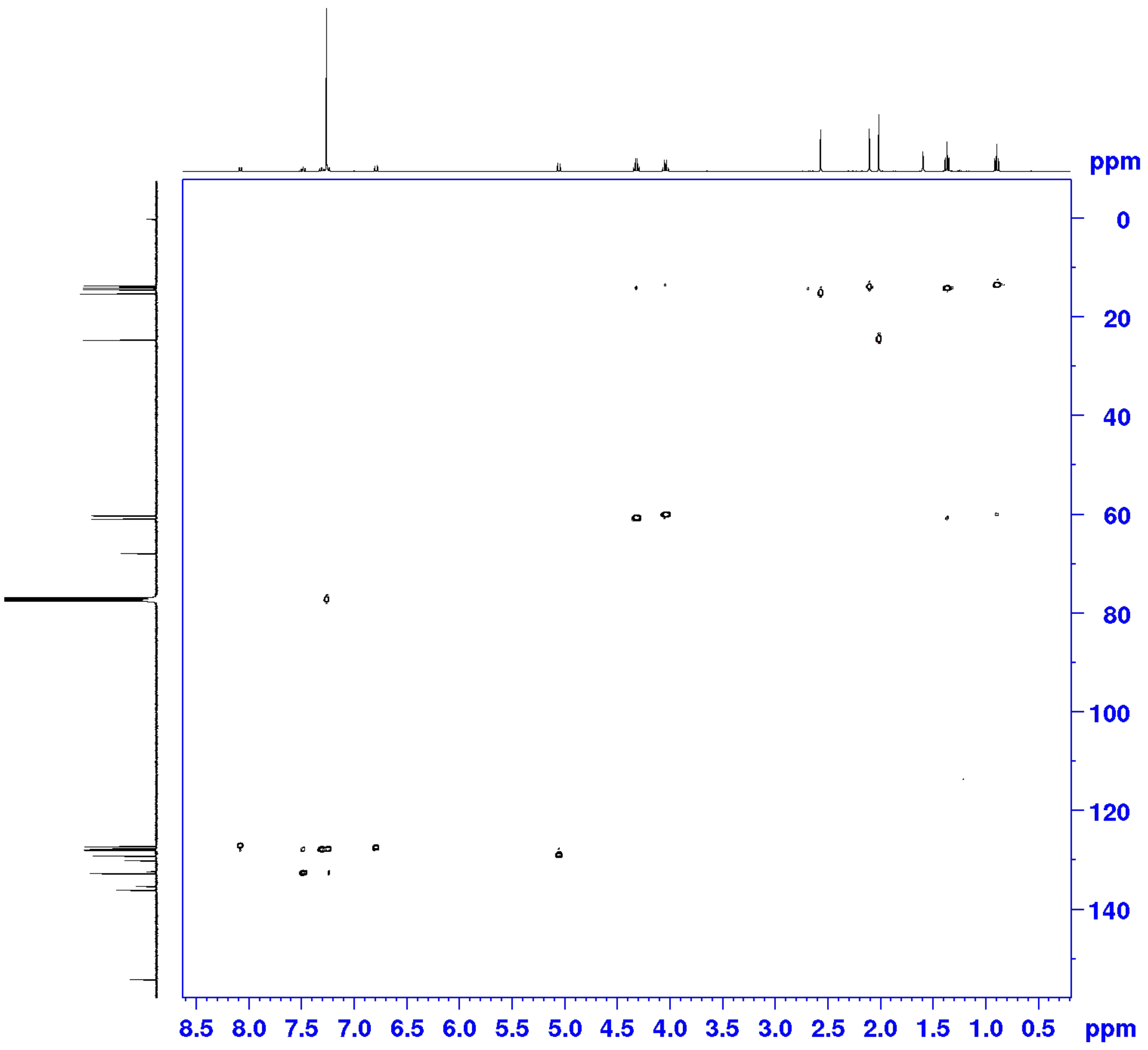

Diethyl (*Z*)-1'-(acetylimino)-3,5-dimethyl-1'*H*-spiro[cyclopentane-1,2'-naphthalene]-2,4-diene-2,4-dicarboxylate (7ca) (HMBC)

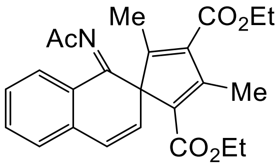

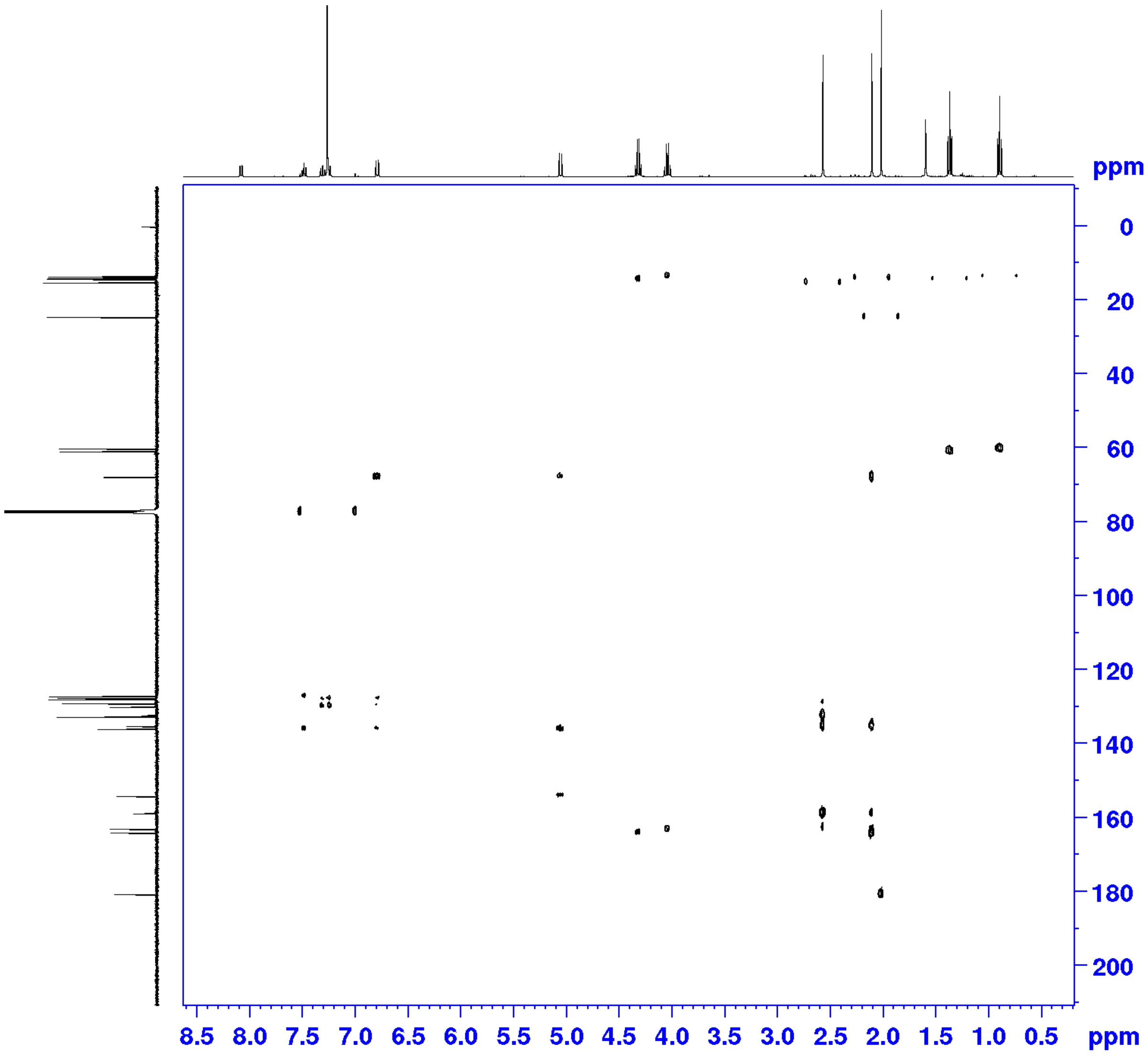

Supplement: Supplementary file 1 [file molecules-23-03325-s001.pdf]
